# Single-cell dissection of remodeled inflammatory ecosystem in primary and metastatic gallbladder carcinoma

**DOI:** 10.1038/s41421-022-00445-8

**Published:** 2022-10-05

**Authors:** Xiang Wang, Chunliang Liu, Jianan Chen, Lei Chen, Xianwen Ren, Minghui Hou, Xiuliang Cui, Youhai Jiang, Erdong Liu, Yali Zong, Anqi Duan, Xiaohui Fu, Wenlong Yu, Xiaofang Zhao, Zhao Yang, Yongjie Zhang, Jing Fu, Hongyang Wang

**Affiliations:** 1 International Cooperation Laboratory on Signal Transduction, National Center for Liver Cancer, Ministry of Education Key Laboratory on Signaling Regulation and Targeting Therapy of Liver Cancer, Shanghai Key Laboratory of Hepato-biliary Tumor Biology, Eastern Hepatobiliary Surgery Hospital, Second Military Medical University, Shanghai, China; 2 Second Department of Biliary Surgery, Eastern Hepatobiliary Surgery Hospital, Second Military Medical University, Shanghai, China; 3 Changping Laboratory, Yard 28, Science Park Road, Changping District, Beijing, China; 4 Research Center for Organoids, The First Affiliated Hospital of Zhengzhou University, Zhengzhou, Henan China; 5 Cancer Research Center, The First Affiliated Hospital of USTC, Division of Life Sciences and Medicine, University of Science and Technology of China, Hefei, Anhui China; 6 School of Life Sciences, Fudan University, Shanghai, China; 7 Second Department of Hepatic Surgery, Eastern Hepatobiliary Surgery Hospital, Second Military Medical University, Shanghai, China

**Keywords:** Cancer microenvironment, Gall bladder cancer

## Abstract

Gallbladder carcinoma (GBC) is the most common biliary tract malignancy with the lowest survival rate, primarily arising from chronic inflammation. To better characterize the progression from inflammation to cancer to metastasis, we performed single-cell RNA sequencing across samples of 6 chronic cholecystitis, 12 treatment-naive GBCs, and 6 matched metastases. Benign epithelial cells from inflamed gallbladders displayed resting, immune-regulating, and gastrointestinal metaplastic phenotypes. A small amount of *PLA2G2A*^+^ epithelial cells with copy number variation were identified from a histologically benign sample. We validated significant overexpression of PLA2G2A across in situ GBCs, together with increased proliferation and cancer stemness in *PLA2G2A*-overexpressing GBC cells, indicating an important role for *PLA2G2A* during early carcinogenesis. Malignant epithelial cells displayed pervasive cancer hallmarks and cellular plasticity, differentiating into metaplastic, inflammatory, and mesenchymal subtypes with distinct transcriptomic, genomic, and prognostic patterns. Chronic cholecystitis led to an adapted microenvironment characterized by MDSC-like macrophages, CD8^+^ T_RM_ cells, and *CCL2*^+^ immunity-regulating fibroblasts. By contrast, GBC instigated an aggressive and immunosuppressive microenvironment, featured by tumor-associated macrophages, Treg cells, CD8^+^ T_EX_ cells, and *STMN1*^+^ tumor-promoting fibroblasts. Single-cell and bulk RNA-seq profiles consistently showed a more suppressive immune milieu for GBCs with inflammatory epithelial signatures, coupled with strengthened epithelial-immune crosstalk. We further pinpointed a subset of senescence-like fibroblasts (*FN1*^+^*TGM2*^+^) preferentially enriched in metastatic lesions, which promoted GBC migration and invasion via their secretory phenotype. Collectively, this study provides comprehensive insights into epithelial and microenvironmental reprogramming throughout cholecystitis-propelled carcinogenesis and metastasis, laying a new foundation for the precision therapy of GBC.

## Introduction

Gallbladder carcinoma (GBC), with adenocarcinoma as the most common histological type, is a relatively rare but stubborn and deadly malignancy worldwide^1^. It commonly develops from gallstones, pancreaticobiliary maljunction (PBM), or seldom gallbladder polyps, usually accompanied by persistent inflammation^2^. GBC carcinogenesis is conceivably concerned by 10%–20% of adults affected by gallstones in the global populations^3^. Nevertheless, unlike gastrointestinal cancers, our understanding of GBC is impeded by the disease rarity and difficulty in acquiring fresh samples, given that biopsy is not always feasible and surgically resected tissues are frequently dedicated to fresh-frozen sections^2^. In a compromise, as one specific subtype, GBC has long been fathomed in a heterogeneous collection of biliary tract cancers across epidemiological, genomic, and clinical studies. In fact, despite resembling other biliary tract cancers in epithelial immunological response or cellular origin, GBC exhibits distinct mucosal structure (e.g., absence of peribiliary glands), physiological function (e.g., vivid fluid transport), and local milieu (e.g., a higher concentration of bile salts), which presumably lead to genomic, transcriptomic, as well as clinical disparities.

Based on accumulated evidence in a century, GBC typifies chronic inflammation-associated cancers (CIACs)^4^. The majority of GBCs reside in a harsh environment besieged by infectious (e.g., pathogens), chemical (e.g., pancreatic juice reflux), mechanical (e.g., gallstone irritation), metabolic (e.g., bile cholesterol supersaturation), and hydrodynamic (e.g., biliary sludge) stresses which, along with heightened cell division stress with aging, contribute to a multitude of microbial, cytotoxic, metabolic, genotoxic, and senescence-related inflammation processes^5^, many of which have initiated and evolved from inflamed gallbladders for ~15 years or more^6^.

In contrast to the complex GBC macro-environment, the tumor microenvironment (TME) knitted by various cell types is likely more intricate. GBC may be driven by environmental and cell-autonomous reprogramming synchronously^7^, yet not fully elucidated by plentiful immunohistochemistry (IHC), flow cytometry, or genomic studies. Although recent bulk RNA sequencing of GBC provides some clues regarding TME compositions^8,9^, the classification system is defined at a low resolution with a mosaic of unsorted cell types. In comparison, single-cell RNA-sequencing (scRNA-seq) will provide a 30,000-foot aerial view to discern niches and networks in the cellular jungle and to discriminate cellular ebb and flow across the initiation, adaptation, and propagation stages of cancers^10^. Two scRNA-seq studies on GBC were only recently published; however, they either focused on GBC of specific genetic subtype^11^, or only had a small sample size^12^, neither delineating the inflammation-cancer-metastasis sequence. Our study profiled and analyzed single-cell transcriptomes from 24 samples across inflamed gallbladders, primary GBCs, and matched metastatic lesions. Tumor heterogeneity, cellular plasticity, and crosstalk across various cell types were depicted. We also explored how the smoldering and adaptive inflammatory milieu was reprogrammed into an elaborately organized pro-tumorigenic ecosystem, notably an aggressive and immunosuppressive TME. This single-cell landscape potentially laid a foundation for decoding enigmas regarding GBC carcinogenesis and metastasis and for uncovering targets of chemo-and immunotherapy.

## Results

### Overview of GBC ecosystem by scRNA-seq

**Fig. 1:**
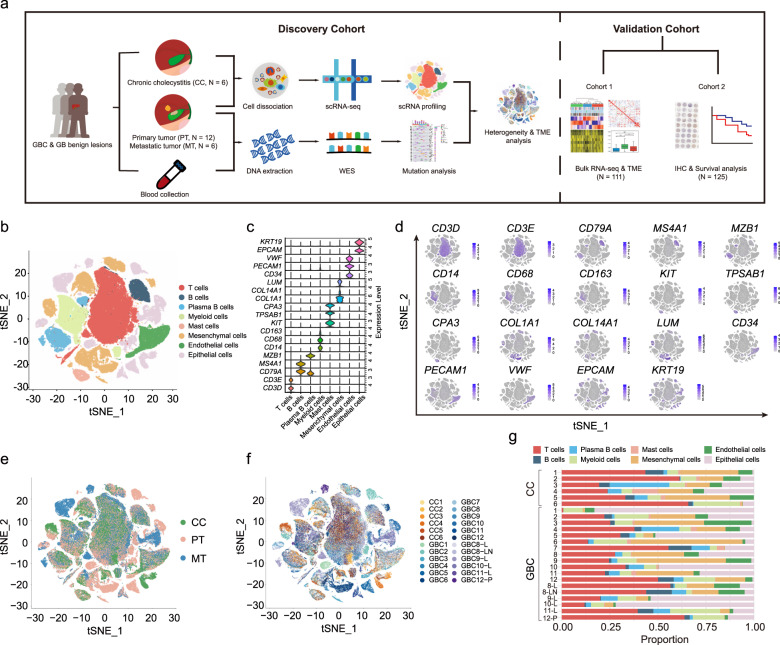
Overview of scRNA-seq analysis across patient-derived gallbladder carcinoma (GBC) samples and non-malignant gallbladder (GB) samples. **a** Workflow for sample collection, processing, sequencing, data analysis, and external validation. **b** *t*-SNE plot visualizing 42 distinct clusters encompassing 140,870 cells from all samples (*n* = 24), colored by cell type (*n* = 8). **c** Violin plots showing expression levels of marker genes for each cell type. **d** *t*-SNE plots visualizing expression levels of cell-type gene signatures among identified cell clusters. **e** *t*-SNE plot visualizing cell clusters colored by tissue of origin. CC chronic cholecystitis; PT primary tumor; MT metastatic tumor. **f** *t*-SNE plot visualizing cell clusters colored by sample. L Liver; LN lymph node; P peritoneum. **g** Horizontal bar charts showing the relative abundance of various cell types in each sample.

We used the droplet-based scRNA-seq platform to profile single cells from 24 surgically resected fresh tissues, including 12 treatment-naive primary tumors of the gallbladder (PT; GBC1–12), 6 matched metastatic tumor samples (MT; four to the liver, one to the lymph node, and one to the peritoneum), and 6 benign gallbladder samples of chronic cholecystitis (CC1–6) (Fig. 1a), confirmed by hematoxylin and eosin (H&E) staining (Supplementary Fig. S1). As a homogeneous adenocarcinoma group, tumor samples spanned a variety of etiologies (gallstone, gallbladder polyps, and PBM), clinical stages (0–IVB), locations (fundus, body, and neck), and histological differentiation grades (poor to good). Benign samples were obtained from distant peritumoral (> 1 cm from the tumor margin), adenomyosis, calculous, PBM-associated, and xanthogranulomatous cholecystitis (XGC)-related gallbladders (Supplementary Table S1).

Following resection, digestion, quality filtering, and doublet removal, we obtained a total of 140,870 cells. We identified 8 cell subpopulations using the *t*-distributed stochastic neighbor embedding (*t*-SNE) method, including T cells (*n* = 50,871; *CD3D*, *CD3E*), B cells (*n* = 5,925; *CD79A*, *MS4A1*), plasma B cells (*n* = 8,490; *CD79A*, *MZB1*), myeloid cells (*n* = 14,958; *CD68*, *CD14*, *CD163*), mast cells (*n* = 1,971; TPSA*B1*, *KIT*, *CPA3*), mesenchymal cells (*n* = 24,680; *COL1A1*, *COL14A1*, *LUM*), endothelial cells (*n* = 8,897; *CD34*, *PECAM1*, *VWF*), and epithelial cells (EPCs, *n* = 25,078; *EPCAM*, *KRT19*) (Fig. 1b–d). EPCs primarily clustered by sample, indicating marked inter-patient heterogeneity, but the stromal and immune cells mainly clustered by cell type with mixed biological origins (Fig. 1e, f). These cell types dispersed among benign and tumor samples to varying extents, and this variation remained for paired lesions from the same case, implying intra- and inter-patient heterogeneity (Fig. 1g; Supplementary Table S2). Generally, compared with CCs, PTs were enriched with more EPCs and mesenchymal cells but with fewer immune cells, especially T cells behaving as tumor-infiltrating lymphocytes (TILs) (Supplementary Fig. S2a, b). These discrepancies implied dynamic cellular adaptation and competition for space and survival amidst different ecosystems^13^.

### Classification of malignant and non-malignant epithelial cells

**Fig. 2:**
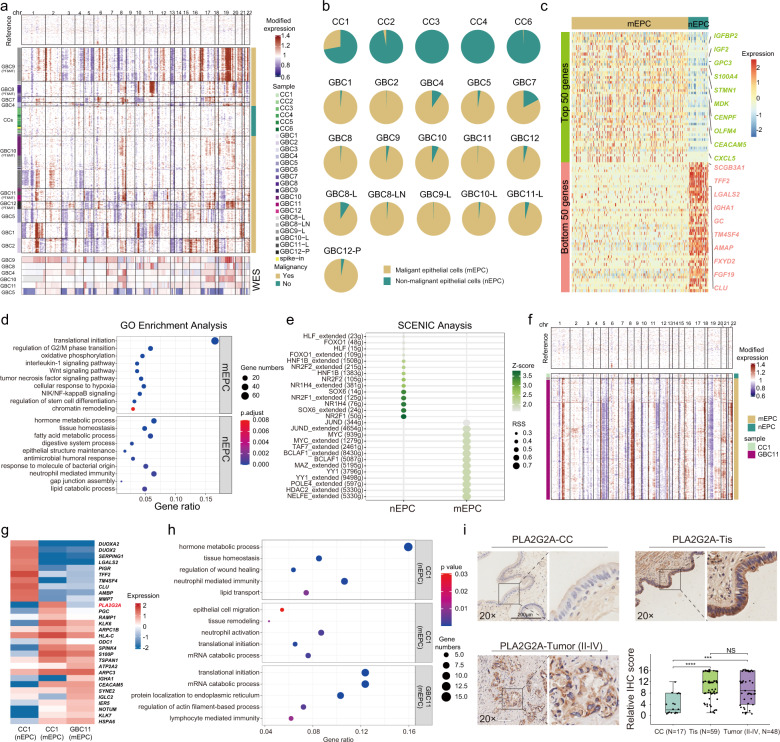
scRNA-seq analysis of malignant epithelial cells (mEPCs) and non-malignant epithelial cells (nEPCs). **a** The landscape of inferred large-scale copy number variations (CNVs) for EPCs among all samples, paired with CNV features revealed by whole-exome sequencing (WES) in six GBC samples. Chromosomal locations are displayed on the top. The left annotation bar indicates sample IDs. The right bar indicates CNV-based categorization of epithelial cells. **b** Pie charts showing the relative abundance of mEPCs and nEPCs across samples with EPC counts ≥ 30 (*n* = 21). **c** Heatmap showing top 50 differentially expressed genes (DEGs) between mEPCs and nEPCs. **d** Dot plots showing differentially enriched GO terms in mEPCs versus nEPCs. **e** Dot plots showing activities of transcription factors among mEPCs versus mEPCs. **f** The landscape of inferred CNVs for EPCs from CC1 and GBC11 (from the same patient), showing a small fraction of cells inferred as mEPCs in CC1. **g** Heatmap showing top-ranking DEGs for CC1-originated nEPCs, CC1-originated mEPCs, and GBC11-originated mEPCs. **h** Dot plots showing significantly enriched GO terms across CC1-originated nEPCs, CC1-originated mEPCs, and GBC11-originated mEPCs. **i** PLA2G2A expression across inflamed gallbladders (*n* = 17), GBCs in situ (Tis; *n* = 59), and GBCs (TNM II-IV; *n* = 48), illustrated by typical IHC staining images (scale bars, 200 μm; 20×) and boxplots (bottom-right) that compared PLA2G2A expression levels between three groups (Wilcoxon rank-sum test; ***P < 0.001, ****P < 0.0001; NS not significant).

Since GBCs harbor enriched copy number variations (CNVs)^14^, we inferred large-scale chromosomal CNVs from the RNA expression profiles to distinguish malignant EPCs (mEPCs) from non-malignant EPCs (nEPCs). Based on the initial cell-type identification, all the EPCs were extracted and inferred for CNVs in each cell, with endothelial cells as the reference and spike-in. After K-means clustering of the CNV profiles, nEPCs were identified as those in the same cluster with spike-in cells and lack of CNVs, predominantly from benign samples. Cells with chromosomal alterations (deletions or amplifications) in other clusters, mostly from GBC samples, were identified as mEPCs (Fig. 2a). Compared with putative benign clusters, the inferred malignant groups exhibited distinct CNV profiles and salient patient occupancy, corresponding to the inter-tumor heterogeneity. The inferred CNVs of mEPCs were consistent with the whole-exome sequencing (WES) data in six cases (Fig. 2a). Overall, 20,644 mEPCs (99.8% from tumor samples) and 3232 nEPCs (81.8% from benign samples) were identified for analyses (excluding samples with cell counts < 30; Fig. 2b; Supplementary Table S2). We next performed bulk RNA-seq analysis of tumoral and peritumoral tissues from GBC7 and used the top 50 differentially expressed genes (DEGs) to construct the tumoral and normal gene set score, respectively. Putative mEPCs defined by inferred CNV in GBC7 exhibited significantly higher tumoral gene set score and lower normal gene set score (Supplementary Fig. S3a, b), verifying the capacity of inferred CNV algorithm in distinguishing DEG-labelled mEPCs.

We next compared DEGs between all the malignant and non-malignant EPCs (Fig. 2c; Supplementary Table S3). Upregulated genes in mEPCs, including *IGF2*, *GPC3*, *S100A4*, *STMN1*, and *MDK*, were semi-automatically assigned to the traditional cancer hallmark framework (Supplementary Table S4)^15^, including proliferative signaling, invasion/metastasis, genomic instability, immune response, and stem cell-like program. We noted a multitude of genes mapping to chromatin remodeling, RNA metabolism, and protein homeostasis. Additionally, mEPCs extensively overexpressed gastrointestinal EPC markers (e.g., *OLFM4*, *PHGR1*) and cell differentiation markers (e.g., *CD9*, *DLK1*), implying the likelihood of gastrointestinal metaplasia^16,17^. These signatures were in line with enriched biological pathways (Fig. 2d) and coincidently exemplified the novel cancer hallmarks published during our peer review process^18^. nEPCs highly expressed conserved cholangiocyte signatures (e.g., *TFF2*, *FGF19*, *MUC5B*) (Fig. 2c), typically engaged in physiological gallbladder functioning, as well as innate or adaptive immune responses against chronic inflammation (Fig. 2d; Supplementary Table S5). Based on SCENIC analysis, nEPCs showed highly active transcription factors (TFs) related to embryogenesis and organogenesis (e.g., *HNF1B*, *NR2F1/2*, *SOX6*) and bile acid metabolism (*NR1H4*).^19^ By contrast, mEPCs displayed oncogenic TF signatures mapping to MYC circuits (e.g., *MYC*, *NELFE*, *HDAC2*) or TP53-related pathways (Fig. 2e; Supplementary Fig. S3c and Table S6).

To further understand the function of nEPCs, these gallbladder cholangiocytes were re-clustered into nine subsets (Supplementary Fig. S3d–f), corresponding to three subtypes: (1) resting cholangiocytes (nEPC01–03), specifically enriched with cholangiocyte signatures (e.g., *SLC26A3*, *FGF19*, *SCGB3A1*, *MUC5B*); (2) immune-regulating cholangiocytes, including nEPC07, principally originated from GBC samples and enriched with interferon-stimulating genes (ISGs), and two clusters mainly from an XGC sample (CC6), namely nEPC04 (*CXCL6*^+^, *IGLC2*^+^, *IGHM*^+^) and nEPC08 (*HLA-DPB1*^+^, *HLA-DQA1*^+^), both showing upregulated epithelial-mesenchymal plasticity (EMP) signatures (*SPP1*^+^, *S100A4*^+^, *VTM*^+^); (3) gastrointestinal metaplastic cholangiocytes, including nEPC05 (enterocyte-like: *FABP1*^+^, *PHGR1*^+^, *REG3A*^+^), mainly derived from PBM-associated GBC10, and two clusters exclusively from PBM-associated CC3, namely nEPC06 (gastric cell-like: *PGC*^+^, *MUC5AC*^+^, *GKN1/2*^+^) and nEPC09 (goblet cell-like: *SPINK4*^+^, *REG4*^+^, *TFF3*^+^)^17,20^. These transcriptomic profiles implied diverse phenotypic plasticity of nEPCs in maintaining mucosal homeostasis and in reacting to inflammation or pancreatic juice reflux stress (Supplementary Fig. S3g).

### Identification of markers for early-stage carcinogenesis of gallbladder epithelia

We unexpectedly identified a small fraction of mEPCs (*n* = 12) with substantial CNV in a benign sample (CC1, the peritumoral tissue of GBC11). Despite some genomic nuances (e.g., fewer copy number gains involving chr8; fewer copy number losses involving chr19), this squad of mEPCs largely shared analogous CNV signatures with the main herd of malignant cells in the matched tumor sample (Fig. 2f). CC1-residing nEPCs showed top-ranking DEGs associated with metabolic process (e.g., *DUOXA2*), gallbladder homeostasis (e.g., *TFF2*), mucosal healing (e.g., *SERPING1*), and inflammatory response (e.g., *PIGR*) (Fig. 2g, h). In contrast, mEPCs from CC1 and GBC11 displayed upregulation of multiple tumor-specific genes (e.g., *S100P*, *TSPAN1*, *CEACAM5*). Putative mEPCs in CC1 and GBC11 shared vivid mRNA translation processes. Dissimilar to enriched pathways in CC1-derived mEPCs (e.g., tissue remodeling, neutrophil activation), mEPCs in GBC11 displayed ectopic expression of *IGHA1* (Fig. 2g), presumably mediating aberrant tumor immunity and promoting tumor aggressiveness (Fig. 2h)^21^. Putative mEPCs in CC1 strikingly showed the highest expression of *PLA2G2A* (log_2_FC = 3.72, *P* = 7.5 × 10^−13^) (Fig. 2g), a typical gastrointestinal mucosal marker^22^. We next interrogated IHC data of our GBC cohort and transcriptomic data of external European Genome-Phenome Archive (EGA) GBC cohort^9^, consistently revealing pronounced overexpression of PLA2G2A among early-stage GBCs. A trend was observed towards reduced PLA2G2A expression among advanced GBCs versus early cases, despite without statistical significance (Fig. 2i; Supplementary Fig. S4c). Moreover, PLA2G2A expression did not seem to correlate well with GBC etiology or overall survival (Supplementary Fig. S4a, b, d). We next established *PLA2G2A*-overexpressing GBC cell lines (NOZ and GBC-SD cells; Supplementary Fig. S4e). *PLA2G2A* overexpression potentiated proliferation, inhibited apoptosis, and significantly facilitated stemness of GBC cells, whereas unexpectedly retarding tumor migration and invasion (Supplementary Fig. S4f–j). Therefore, *PLA2G2A* likely served a stage-dependent role, more actively engaged in early carcinogenesis than in tumor progression.

### Identification of diverse subtypes of mEPCs associated with GBC prognosis

**Fig. 3:**
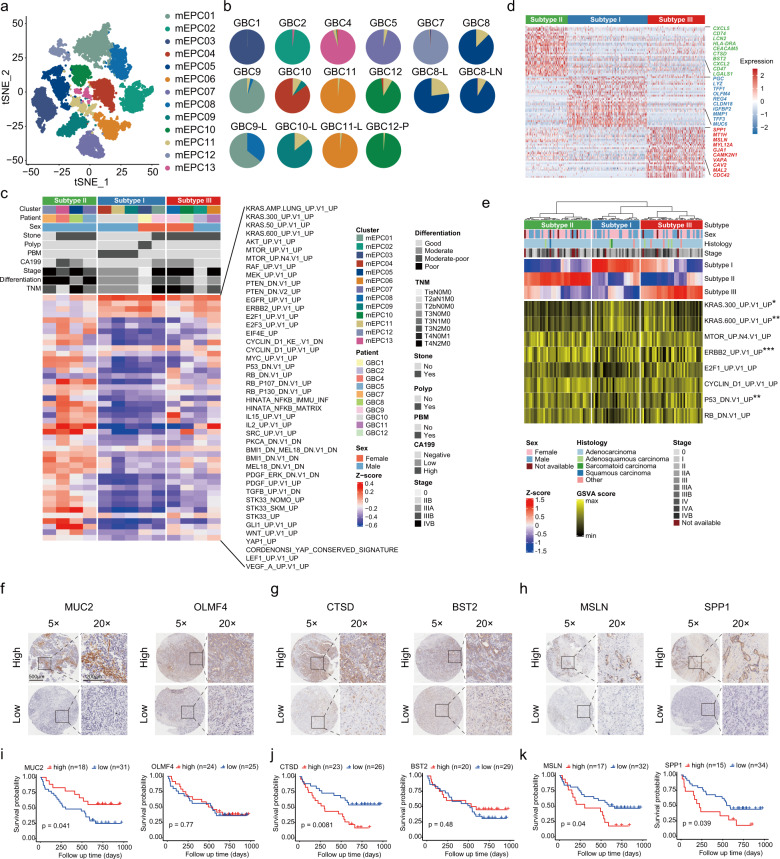
Characterization of three subtypes of mEPCs based on 10× scRNA-seq. **a** *t*-SNE plot showing mEPCs colored by clusters (*n* = 13). **b** Pie charts showing the relative abundance of mEPC clusters (colored in line with **a**) across tumor samples (*n* = 16, excluding two samples with EPC counts < 30). **c** Heatmap showing three subtypes of mEPCs classified by hierarchical clustering based on GSVA analysis (MSigDb_C6 oncogenic signatures), with corresponding clinicopathological features displayed. **d** Heatmap showing top-ranking DEGs for three subtypes of mEPCs. **e** Heatmap showing the assignment of scRNA-seq-defined mEPC subtypes by analyzing the bulk RNA-seq data from an external GBC cohort (*n* = 111), based on GSVA analysis (MSigDb_C6 oncogenic signatures). *P* values for pathways with a significant enrichment are displayed (one-way ANOVA test; **P < 0.01, ***P < 0.001). **f–h** Representative IHC staining images of markers for subtype I (MUC2, OLMF4), subtype II (CTSD, BST2), and subtype III (MSLN, SPP1) by using our GBC tissue microarray cohort (*n* = 49), respectively. Scale bars, 500 μm, 5×; 200 μm, 20×. **i–k** Kaplan–Meier curves of overall survival when stratifying patients by high or low expression of mEPC subtype markers. *P* values were calculated by log-rank test.

We identified 13 subsets of mEPCs mostly clustered by patient (Fig. 3a; Supplementary Fig. S5a), implying inter-tumoral heterogeneity. Several samples harbored mixed clusters, suggesting coexisting intra-tumoral heterogeneity (Fig. 3b). Most clusters exhibited distinct transcriptomic profiles; however, those from the same patient (e.g., mEPC01 and 08 from GBC9) displayed overlapping transcriptomic profiles to varying extents (Supplementary Fig. S5b, c). Inferred CNV signatures for each cluster were in line with distinct or shared transcriptomic traits (Supplementary Fig. S5d). For instance, underlying the extensive copy number gains at chr19 for mEPCs, 28% of the top 100 cancer-related DEGs were found at chr19q13 (Supplementary Table S4).

Gene set variation analysis (GSVA) revealed three distinct mEPC subtypes based on MSigDb_C6 signatures. Subtype I (metaplastic mEPCs) exhibited enrichment of KRAS-related circuits and highly expressed enterocyte-like (*REG4*, *OLFM4*, *MUC2*) or gastric cell-like (*LYZ*, *PGC*, *MUC6*) markers (Fig. 3c, d; Supplementary Table S7), implying intestinal and/or pseudopyloric metaplasia. Related patients (GBC1, 9, 10) generally had an earlier stage of disease and good-to-moderate differentiation associated with gallbladder polyps or PBM. Subtype II (inflammatory mEPCs) exhibited enriched inflammatory pathways (e.g., NF-κB, IL-2, IL-15) and DNA damage-related pathways (e.g., P53, RB, MYC) (Fig. 3c), reminiscent of the exacerbated tumor-promoting DNA damage induced by chronic inflammation^23^. Plentiful immune or inflammatory marker genes (e.g., *CXCL5, CTSD, BST2, CD47*) were identified (Fig. 3d). Most related cases (GBC4, 5, 7, 8) had gallstones and suffered an advanced stage with poor differentiation and lymph node metastasis. As for subtype III (mesenchymal mEPCs; GBC2, 9, 11, 12), in addition to upregulated KRAS-related pathways, we noted activated SRC and Wnt pathways, both serving as master regulators of cell-cell adhesion and EMP^24,25^, and typical marker genes associated with cell migration (e.g., *GJA1*, *MT1H*) and EMP (e.g., *SPP1, MSLN*) (Fig. 3c, d). IHC analysis further validated increased protein levels of subtype-specific markers in corresponding GBC patients, including *MUC2*, *CTSD*, and *MSLN* for subtypes I, II, and III, respectively (Supplementary Fig. S6a–c). Based on subtype-specific single-cell transcriptomic signatures, we calculated a reference matrix for deconvolution analysis of the bulk RNA-seq data in the EGA GBC cohort (*n* = 111). The decomposition analysis of this external validation cohort revealed three phenotypes akin to our classification system, with more early-stage GBCs in subtype I. In line with our findings, enriched KRAS-related signaling was present for subtypes I and III but not for subtype II, which conversely showed enriched ERBB2 and P53 pathways (Fig. 3e).

The association between subtype-specific markers and prognosis was explored in another independent GBC cohort from our hospital based on tissue microarray data (*n* = 49; Fig. 3f–h; Supplementary Table S8). Increased expression of MUC2 (subtype I marker) was markedly associated with better overall survival (*P* = 0.041; Fig. 3i). By contrast, overexpression of CTSD (subtype II marker), MSLN (subtype III marker), or SPP1 (subtype III marker) likely predicted decreased overall survival (*P* = 0.008, *P* = 0.040, and *P* = 0.039, respectively; Fig. 3j, k). We further jointly used these markers to distinguish GBC subtypes: *MUC2*^high^*CTSD*^low^*MSLN*^low^ subtype_I, *CTSD*^high^*MUC2*^low^*MSLN*^low^ subtype_II, and *MSLN*^high^*MUC2*^low^*CTSD*^low^ subtype_III. Compared with subtype_I, both subtype_II and subtype_III GBCs had substantially decreased overall survival (Supplementary Fig. S6d). Taken together, inflammatory and mesenchymal signatures of mEPCs were more closely associated with GBC aggressiveness.

Moreover, the somatic mutation landscape based on available WES data showed that subtype_II GBC samples altogether displayed *TP53* mutation and high tumor mutational burden (TMB), which partly accounted for the activated immune signatures (Supplementary Fig. S7a)^26^. *KRAS* mutation was captured in a subtype_I sample (GBC1), paralleling the GSVA findings, reminiscent of the role of *KRAS* mutation in the gallbladder metaplasia-cancer sequence^27^. We successfully established two different organoids (GBO-819 and GBO-831) from the benign human gallbladder mucosa, both showing non-dysplastic CK7^+^ epithelial structure. Unlike GBO-819 (MUC2^−^REG4^−^), MUC2^+^REG4^+^ GBO-831 represented the intestinal metaplastic organoid (Supplementary Fig. S7b). The introduced *KRAS*^G12D^ mutation did not lead to dysplastic alterations in GBO-819 (Supplementary Fig. S8). By contrast, the metaplastic organoid GBO-831 displayed malignant transformation of the gallbladder epithelium after *KRAS*^G12D^ introduction, evidenced by the altered nucleo-cytoplasmic ratio, nuclear atypia, and mitosis. Further administration of *KRAS*^G12D^ inhibitor 14 markedly relieved GBO-831 from oncogenic transformation (Supplementary Fig. S9). These data strongly suggested a crucial role of KRAS signaling in the tumorigenesis of metaplastic gallbladder epithelia.

We next compared transcriptomic profiles of mEPCs between paired primary and metastatic samples from five patients respectively. Three patients (GBC8, 11, 12) displayed intermingled mEPCs; however, the other two cases (GBC9, 10) showed clearly demarcated metastasis-specific clusters (Supplementary Fig. S10a). Despite broad similarities in CNV signatures, a few distinct genomic variations were shown for metastatic versus primary tumors (e.g., chr12 amplification in GBC9-MT; chr2 amplification in GBC10-MT) (Supplementary Fig. S10b). In both cases, metastatic mEPCs exhibited substantially boosted translational initiation and ribosome biogenesis, mirrored by the upregulated genes (e.g., *DDX1* and *CENPW* in GBC9-MT; *RPL31* and *RPS18* in GBC10-MT) (Supplementary Fig. S10c–e). Intriguingly, we noted several candidate metastasis-promoting biomarkers potentially serving as anti-cancer targets. For instance, GBC9-MT showed elevated expression of *CD24* (immune checkpoint in promoting immune evasion) and *IGFBP2* (metabolic checkpoint in promoting tumor growth), and GBC10-MT displayed cancer stem cell signature (*OLFM4*) and enhanced cell migration (e.g., *CLDN3*, *CEACAM5*) (Supplementary Table S6).

These results together revealed three subtypes of gallbladder cancer cells relevant to patient prognosis, which displayed distinct transcriptomic and genomic signatures, providing potential markers and targets for GBC diagnosis and treatment.

### Identification of myeloid cell subsets nurturing the immunosuppressive TME

**Fig. 4:**
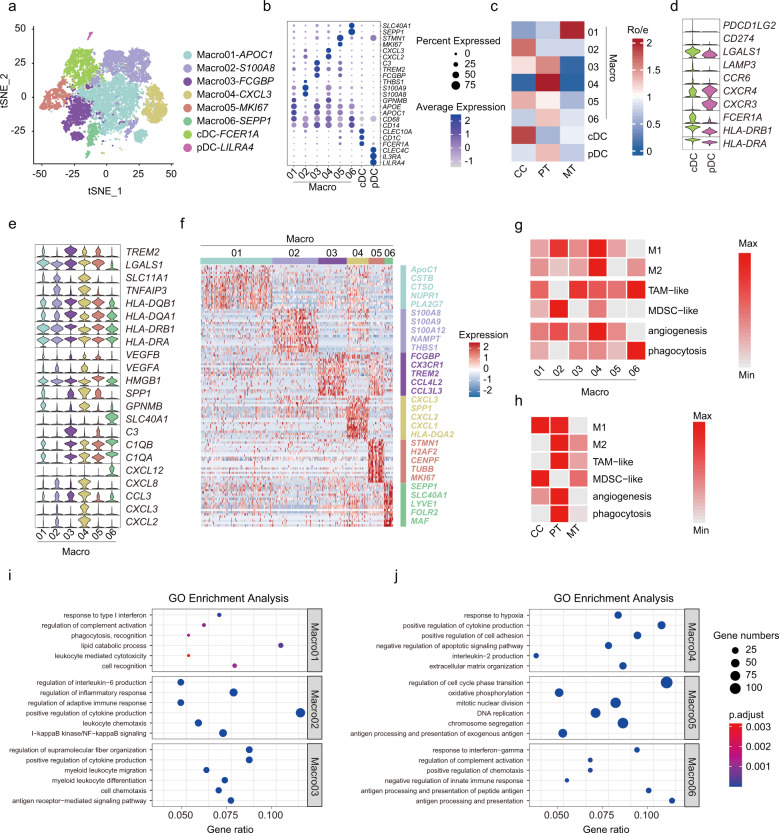
Characterization of myeloid cells by 10× scRNA-seq. **a** *t-*SNE plot showing eight color-coded myeloid cell clusters, including six macrophage clusters and two dendritic cell clusters. **b** Bubble heatmap showing expression levels of marker genes across macrophage and dendritic cell subsets. **c** Heatmap showing preferential enrichment of eight myeloid cell clusters across CCs, PTs, and MTs. Ro/e > 1 indicates significant enrichment. **d** Violin plot showing expression of representative gene signatures among dendritic cell subsets. **e** Violin plots showing expression of functional gene sets across macrophage clusters (from right top to bottom: innate host defenders against microbes; HLA molecules; markers for angiogenesis and hypoxia; complement components; inflammatory chemokines). **f** Heatmap showing top-ranking DEGs for each macrophage subset. **g** Heatmap displaying functional assignment of each macrophage cluster. TAM, tumor-associated macrophage; MDSC, myeloid-derived suppressor cells. **h** Heatmap showing the relative abundance of functional macrophage subsets across CCs, PTs, and MTs. **i**, **j** Dot plots showing typically significantly enriched GO terms across macrophage clusters.

Innate myeloid cells are at the vanguard of host defense against tumor initiation. Tumor-infiltrating myeloid cells play essential roles in regulating immune responses and facilitating cancer progression^28^. We re-clustered all myeloid cells (*n* = 13,873) and identified eight distinct subsets, including macrophages (Macro01-06), conventional dendritic cells (cDCs; *FCER1A*^+^), and plasmacytoid dendritic cells (pDCs; *LILRA4*^+^) (Fig. 4a, b; Supplementary Table S9).

Macro01 was preferentially enriched in metastatic lesions (Fig. 4c; Supplementary Fig. S11a) and was mapped to tumor-associated macrophages (TAMs) (Fig. 4g; Supplementary Table S10), with extensive upregulation of ISGs (e.g., *ISG15*, *GPNMB*, *IFI6*) and lipid metabolism genes (e.g., *APOC1*, *CTSD*, *PLA2G7*) (Fig. 4b, f; Supplementary Table S9), conceivably implying its immunosuppressive role conferred by IFN-stimulated lipid reprogramming^29,30^. The *S100A8*^+^*THBS1*^+^ Macro02 subset, rich in S100A family genes (*S100A8*, *S100A9*, *S100A12*, *VCAN*, and *FCN1*), typically mapped to myeloid-derived suppressor cell-like (MDSC-like) macrophages (Fig. 4f, g)^31–33^. Apart from its affluence in inflamed gallbladders akin to other chronic infections (Fig. 4c)^34^, several GBC patients, especially one advanced case (GBC9), showed pronounced enrichment (Supplementary Fig. S11a), whereby this subset conceivably induced an immunocompromised state through regulating cytokine production and leukocyte chemotaxis (Fig. 4i). Macro03 (*FCGBP*^+^*CX3CR1*^+^*C3*^*+*^) was enriched in GBCs (Fig. 4c), especially in two early cases (GBC1-2) (Supplementary Fig. S11a), typically expressing the tumor-promoting marker *TREM2* (Fig. 4b, e, f)^35^. It behaved as an immunosuppressive TAM subset by regulating leukocyte migration, differentiation, and chemotaxis (Fig. 4i). With marked patient occupancy (GBC6) (Fig. 4c; Supplementary Fig. S11a), Macro04 exhibited versatile tumor-promoting roles, including cytokine production (e.g., *TNFAIP3*, *CXCL3*), pro-angiogenesis (e.g., *VEGFA*), and ECM remodeling (e.g., *SPP1*) (Fig. 4e–g, j)^28,36,37^. Macro05 displayed prominently active proliferation features (*MKI67*^+^*STMN1*^+^) (Fig. 4b, f), likely serving as self-renewal gallbladder-resident macrophages^38^. This subcluster was frequently found within GBCs (Supplementary Fig. S11a); however, a benign outlier (CC6 with XGC) was noted, whereby proliferating macrophages probably contributed to the shaping of ‘foamy’ macrophage milieu in XGC-related destructive inflammation. Predominantly residing in PTs (Fig. 4c), Macro06 synchronously behaved as M2-like TAMs (e.g., *LYVE1*, *SEPP1*, *MRC1*, *FOLR2*)^39^, as well as perivascular TAMs (e.g., *MRC1*, *VCAM1*, *SLC40A1*) (Fig. 4f, g), which probably facilitated vascular development and cancer cell intravasation (Fig. 4c–g)^32,40^. Collectively, tumor-derived macrophages displayed more prominent M2- and TAM-like traits, together with boosted angiogenesis and phagocytosis processes. In contrast, their benign counterparts behaved more like M1- or MDSC-like macrophages (Fig. 4h). As for different epithelial subtypes, subtype II exhibited more active crosstalk with macrophages (Supplementary Fig. S11b). Cancer cell-derived MIF or COPA potentially dictated the crosstalk with macrophages through CD74-related signaling pathways and, reciprocally, macrophage-secreted granulin (GRN) might send tumor-promoting signals to cancer cells via TNF receptors (Supplementary Fig. S11c, d).

Dendritic cells (DCs) consisted of type 1 conventional DCs (cDC1s), type 2 cDCs (cDC2s) and plasmacytoid DCs (pDCs)^41^. We identified cDC2s (*CD1C*^+^*FCER1A*^+^) as the predominant cDC subset (Fig. 4a), paralleling with previous pan-cancer single-cell data^28,42^. The cDC subset displayed prominent antigen presentation properties (*HLA-DRA*^high^*HLA-DRB1*^high^), whereas pDCs extensively expressed chemotactic receptors (*CXCR3*^high^*CXCR4*^high^) (Fig. 4d). The cDC subset seemed to be independent of histological origin, implying a general naive T-cell priming program shared by inflamed gallbladders and GBCs (Supplementary Fig. S11a). By contrast, despite being less abundant (*n* = 89), the pDC subset chiefly resided in tumors (Fig. 4c), serving as a multitasking player in creating the immunosuppressive milieu, such as T-cell proliferation suppression (*GZMB*)^43^, interferon production inhibition (*PTPRS*)^44^, and metabolic rewiring (*CLIC3*) (Supplementary Table S9)^45^. Collectively, these results implied a pivotal role of myeloid cell subsets in nurturing the immunosuppressive gallbladder TME.

### T cell phenotypes reveal regulators modulating immune evasion in GBC

**Fig. 5:**
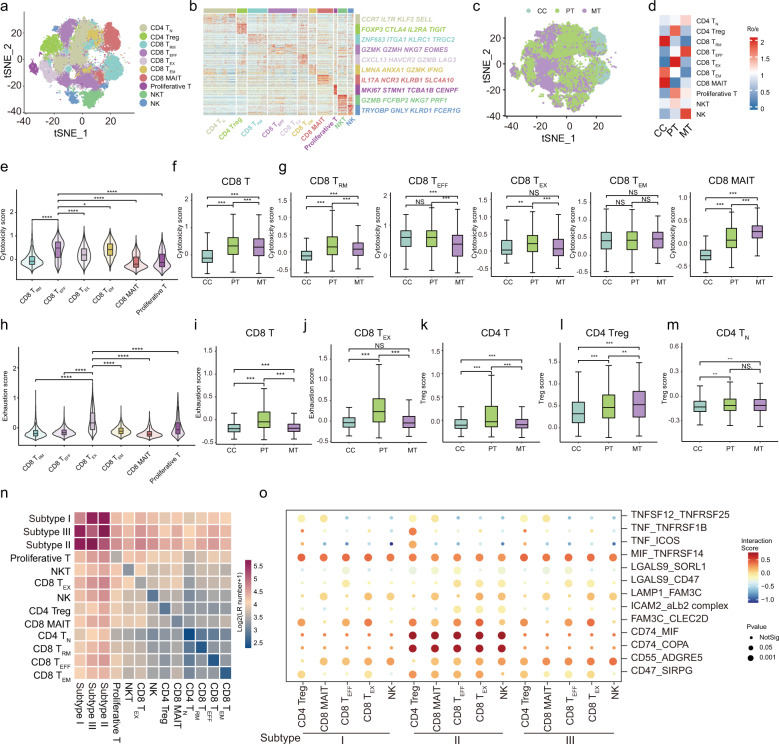
Clustering and functional analysis of T Cells and NK cells. **a** *t*-SNE plot showing color-coded clusters of T cells (*n* = 8) and NK cells (*n* = 2). T_N_, naive T cells; Treg, regulatory T cells; T_RM_, tissue-resident memory T cells; T_EFF_, effector T cells; T_EM_, effector memory T cells; MAIT, mucosal-associated invariant T cells. **b** Heatmap showing transcription levels of typical gene signatures across different T cell and NK cell subsets. **c** *t*-SNE visualization of T cells and NK cells colored by tissue of origin (CCs, PTs, or MTs). **d** Heatmap showing the preferential enrichment of T cell and NK cell clusters across CCs, PTs, and MTs. Ro/e > 1 indicates significant enrichment. **e** Violin plot displaying cytotoxicity scores for CD8^+^ T cell subsets, by comparing CD8^+^ T_EFF_ cells with other CD8^+^ T cell clusters. *P < 0.05; ****P < 0.0001. **f** Boxplots comparing cytotoxicity scores for CD8^+^ T cells between CCs, PTs, and MTs. ***P < 0.001. **g** Boxplots comparing cytotoxicity scores for CD8^+^ T_RM_, CD8^+^ T_EFF,_ CD8^+^ T_EX_, CD8^+^ T_EM,_ and CD8^+^ MAIT cell subsets between CCs^,^ PTs, and MTs^,^ respectively. NS, no significance; **P < 0.01; ***P < 0.001. **h** Violin plot showing exhaustion scores for CD8^+^ T cell subsets, by comparing CD8^+^ T_EX_ cells with other CD8^+^ T cell subsets. ****P < 0.0001. **i** Boxplots comparing exhaustion scores for CD8^+^ T cells between CCs, PTs, and MTs. ***P < 0.001. **j** Boxplots comparing exhaustion scores for CD8^+^ T_EX_ cells between CCs, PTs, and MTs. ***P < 0.001; NS, no significance. **k–m** Boxplots comparing regulatory T (Treg) scores for CD4^+^ T cells, CD4^+^ Treg cells, and CD4^+^ T_N_ cells between CCs, PTs, and MTs, respectively. **P < 0.01; ^*^**P < 0.001; NS, no significance. **n** Heatmap showing the strength of potential ligand-receptor (LR) interactions between mEPC subtypes and T cell/NK cell subsets predicted by CellphoneDB. **o** Bubble plot showing representative ligand-receptor pairs between mEPC subtypes and T cell/NK cell subsets. Dot size indicates *P* value, colored based on interaction score. *P* values were calculated by Wilcoxon rank-sum test for **e–m**.

TILs are an integral component of the GBC microenvironment and potentially predict prognosis and therapy response^46^. By clustering T cells and natural killer (NK) cells (*n* = 50,871; Supplementary Table S11), we identified ten major subsets: (1) CD4^+^ T cell subsets (*n* = 2), including naive T (T_N_) and regulatory T (Treg) cells; (2) CD8^+^ T cell subsets (*n* = 6), including resident memory T (T_RM_), effector T (T_EFF_), exhausted T (T_EX_), effector memory T (T_EM_), mucosal-associated invariant T (MAIT), and proliferative T cell subsets; (3) NK cell subsets (*n* = 2), including conventional NK and NKT cells (Fig. 5a). Each subset was annotated by canonical marker genes (Fig. 5b; Supplementary Fig. S12a). These immune cell clusters were distributed across inflamed, primary, and metastatic samples to varying extents; however, histologically different samples exhibited preferential enrichment (Fig. 5c, d; Supplementary Fig. S12b–d). Chronic cholecystitis was rich in CD8^+^ MAIT and memory T cell subsets (T_RM_ and T_EM_). In contrast, other immune cell types were chiefly enriched in GBCs. Specifically, PTs were mostly enriched with CD4^+^ Treg, CD8^+^ T_EX_, and CD8^+^ proliferative T cells, whereas metastatic lesions were more infiltrated with CD4^+^ T_N_, CD8^+^ T_EFF_, and NK cells (Fig. 5d; Supplementary Fig. S12b). We further quantitatively compared the cytotoxicity, exhaustion, and Treg scores between cell types and between sample types (Supplementary Table S12). Compared with other CD8^+^ T cells, CD8^+^ T_EFF_ cells showed distinctly higher cytotoxicity scores (Fig. 5e). CD8^+^ TILs in MTs had a lower cytotoxicity score than those in PTs, especially CD8^+^ T_EFF_ and CD8^+^ T_RM_ cells (Fig. 5f, g). CD8^+^ TILs within PTs, particularly the CD8^+^ T_EX_ subset, showed higher exhaustion scores than those in CCs or MTs (Fig. 5h–j). Tumor-infiltrating CD4^+^ T cell populations, including CD4^+^ Treg and CD4^+^ T_N_ subsets, showed markedly increased Treg scores than those residing in CCs (Fig. 5k–m). These results reflected the abnormal distribution and dysfunctional state of T cells in GBC TME, providing a resource for further investigation of GBC immunotherapy.

We next investigated interactions between mEPCs and T or NK cells. Compared to subtypes I and III, subtype II EPCs exhibited strengthened crosstalk with various lymphocyte subsets (Fig. 5n, o). mEPCs communicated with T cells most extensively via MIF-TNFRSF14 (HVEM) interactions, followed by FAM3C-CLEC2D and CD55-ADGRE5 pairs. mEPCs also interacted with immunosuppressive T cells (CD4^+^ Treg and CD8^+^ T_EX_ cells) through ligands CD47 and LGALS9, whereby CD47-related ‘do not eat me’ signals and LGALS9-triggered inhibitory pathways presumably facilitated GBC immune evasion (Supplementary Table S6). Meanwhile, immune checkpoints *LAG3* and *HAVCR2* (encoding receptor for *LGALS9*) were highly expressed in CD8^+^ T_EX_ cells (Supplementary Table S11), suggesting the presence of functional crosstalk between mEPCs and CD8^+^ T_EX_ cells. These significant interactions presumably contributed to an immunosuppressive TME and represented appealing immune checkpoints for therapeutic targeting.

### Characterization of innate and adaptive immune cell landscape associated with EPC phenotypes

**Fig. 6:**
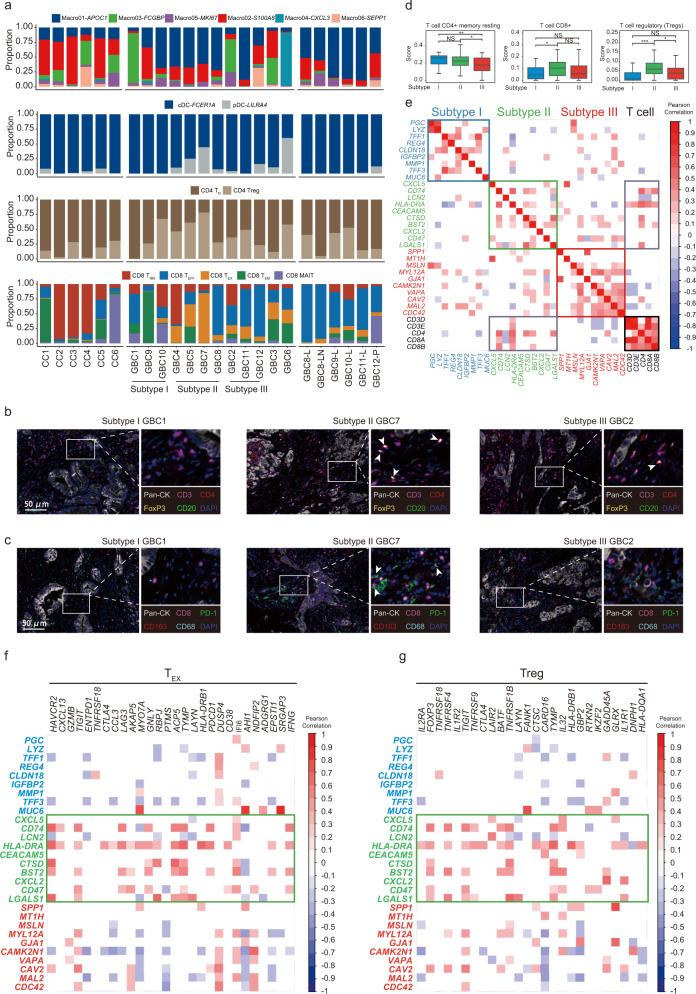
Landscape of innate and adaptive immune microenvironment. **a** Bar charts showing the relative abundance of various immune cell subsets (from top to bottom: macrophages, DCs, CD4^+^ T cells, and CD8^+^ T cells) in each sample of our scRNA-seq cohort. **b, c** Representative immunofluorescence staining for epithelial markers (pan-CK) and immune cell markers (CD3, CD4, CD8, CD20, CD163, PD-1, and FoxP3) across samples of different mEPC subtypes. Scale bars, 50 μm. CD4^+^FoxP3^+^ cells are highlighted by arrows in **b**, and arrows in **c** indicate CD8^+^PD-1^+^ cells. **d** Boxplots comparing CIBERSORT scores of typical T cell subsets between differentially assigned subtypes of GBCs, based on external bulk RNA-seq data (*n* = 111). **e** Correlation matrix showing the relationship between mEPC subtype gene signatures and T cell marker genes, based on external bulk RNA-seq data (*n* = 111). **f**, **g** Correlation matrix showing the relationship between mEPC subtype gene signatures and CD8^+^ T_EX_ cell marker genes (**f**) or CD4^+^ Treg marker genes (**g**), based on external bulk RNA-seq data (*n* = 111).

We next systematically depicted the sample-specific landscape (e.g., distribution, dynamic variations) of innate and adaptive immune cell populations categorized by histological types, disease stages, and EPC-based phenotypes (Fig. 6a). Chronic cholecystitis and malignant samples were enriched with MDSC-like macrophages (Macro02) and immunosuppressive TAMs (e.g., Macro01), respectively. Compared with inflamed lesions, malignant samples displayed an overall more immunosuppressive state, typified by expanding pDCs, CD4^+^ Tregs, and CD8^+^ T_EX_ cells, along with decreased memory T cells. We further cemented these disparities either by comparing matched peritumoral (CC1) and tumor (GBC11) lesions from the same patient or by comparing inflamed (CC3) and cancerous (GBC10) lesions with the same PBM etiology. Moreover, each mEPC subtype exhibited a distinct pattern in recruiting innate and adaptive immune cells. Strikingly, inflammatory subtype II GBCs harbored a more suppressive TME with higher proportions of pDCs, Tregs, and CD8^+^ T_EX_ cells. Multiplex immunofluorescence staining further validated that CD8^+^PD-1^+^ T_EX_ and CD4^+^FOXP3^+^ Treg cells were more frequently seen in subtype II GBC versus other subtypes (Fig. 6b, c). We next referred to the external EGA bulk RNA-seq dataset to quantitatively interrogate the preferential enrichment of T cells across differentially assigned GBC subtypes. Compared to the other two types, subtype II GBCs displayed the highest proportion of CD8^+^ T and CD4^+^ Treg subsets (Fig. 6d). Further pairwise correlation analyses revealed a closer correlation between subtype II markers (e.g., *CD74*, *CTSD*, *CD47*, *LGALS1*) and various T cell markers (e.g., *CD3D*, *CD4*, *CD8A*; Fig. 6e). Specifically, we noted a positive correlation between subtype II markers and representative CD8^+^ T_EX_ or CD4^+^ Treg gene sets (Fig. 6f, g). Collectively, these results suggested that epithelial cell phenotype might remodel the immunologic microenvironment, which in turn expedited tumor progression.

### Dissection of mesenchymal components and regulators underlying GBC progression

**Fig. 7:**
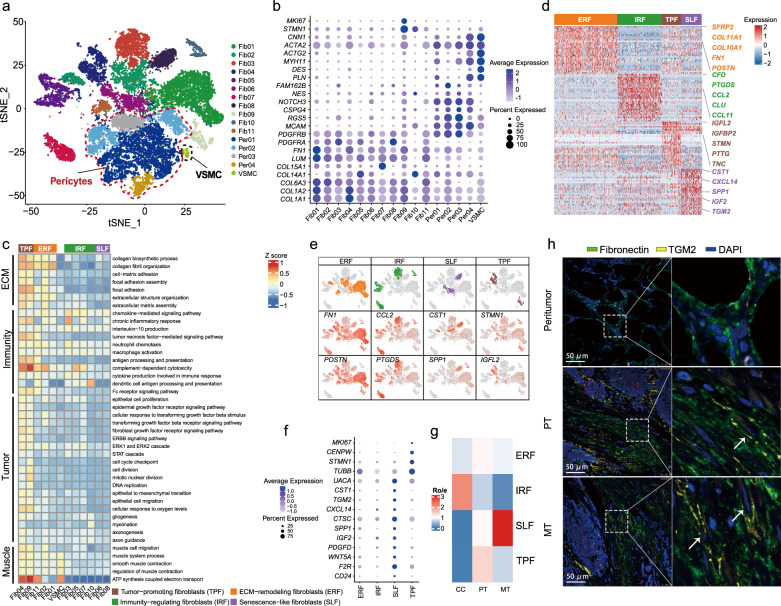
Mesenchymal microenvironment of GBCs. **a** *t*-SNE plot visualizing 16 clusters across three major compartments of mesenchymal cells, including fibroblasts, pericytes, and VSMCs. VSMC, vascular smooth muscle cell. **b** Bubble heatmap showing marker genes across mesenchymal clusters. Dot size indicates the percentage of positively expressed cells, colored by the expression level. **c** Heatmap showing four phenotypic subtypes of fibroblasts with differentially enriched GO terms, based on GSVA analysis. Subtype names are annotated at the bottom right. **d** Heatmap showing typical DEGs across four fibroblast subsets. **e** *t*-SNE plots visualizing color-coded expression of marker genes across four fibroblast subsets. **f** Bubble plot showing gene signatures for four fibroblast subtypes. **g** Heatmap showing the preferential enrichment of different fibroblasts subtypes across CCs, PTs, and MTs. Ro/e > 1 indicates significant enrichment. **h** Representative immunofluorescent staining of senescence-like fibroblast (Fibronectin^+^ TGM2^+^) in peritumoral, PT, and MT lesions from the same GBC patient. Scale bars, 50 μm; white arrow, Fibronectin^+^TGM2^+^ fibroblasts.

GBC cells are embedded in a desmoplastic stromal microenvironment^8^. We identified three major mesenchymal cell types, including fibroblasts (Fib01-Fib11; *COL6A3*^+^, *PDGFRA*^+^, *FN1*^+^), pericytes (Peri01-Peri04; *RGS5*^+^, *CSPG4*^+^, *MCAM*^+^), and vascular smooth muscle cells (VSMCs; *MYH11*^+^, *ACTA2*^+^, *DES*^+^) (Fig. 7a, b). The pathway activation and related gene expression were further assessed to explore the diverse phenotypes of fibroblasts (Fig. 7c–g; Supplementary Table S13). Three clusters (Fib01, 02, and 11) were engaged in collagen biosynthesis and organization with high expression of *COL11A1, FN1*, and *POSTN*, implying a phenotype of ECM-remodeling fibroblasts (ERF). Four fibroblast clusters (Fib03, 05, 07, and 10), primarily inhabiting inflamed samples (Fig. 7g; Supplementary Fig. S13a, b), were actively involved in inflammatory responses, chemokine signaling, and complement activation (e.g., *CCL2*, *PTGDS*, *CFD*), indicating a phenotype of immunity-regulating fibroblasts (IRF). Two tumor-originated clusters (Fib04, Fib09) showed distinctive enrichment in cell-cycle (e.g., *STMN, PTTG*) and TNFα signaling pathway (Fig. 7c–f), typically co-opting the wound healing-like programs of cellular proliferation and metabolic rewiring (e.g., *IGFL2, TNC*) (Fig. 7d)^47^. Hence, they were labeled as tumor-promoting fibroblasts (TPF). Fib06 and Fib08 were enriched in tumor tissues, especially metastatic lesions (Fig. 7g; Supplementary Table S13), exhibiting proliferation quiescence (*MKI67*^−^*CENPW*^−^) and enhanced secretory capacity (e.g., *CST1, CXCL14, TGM2, SPP1, IGF2*) (Fig. 7d–f), corresponding to the senescence-associated secretory phenotype (SASP)^48^. As such, we termed them as senescence-like fibroblasts (SLF). The senescence-like fibroblasts also highly expressed *F2R* and *CD24* (Fig. 7f), implying a role in suppressing anti-tumor immunity^49,50^.

**Fig. 8:**
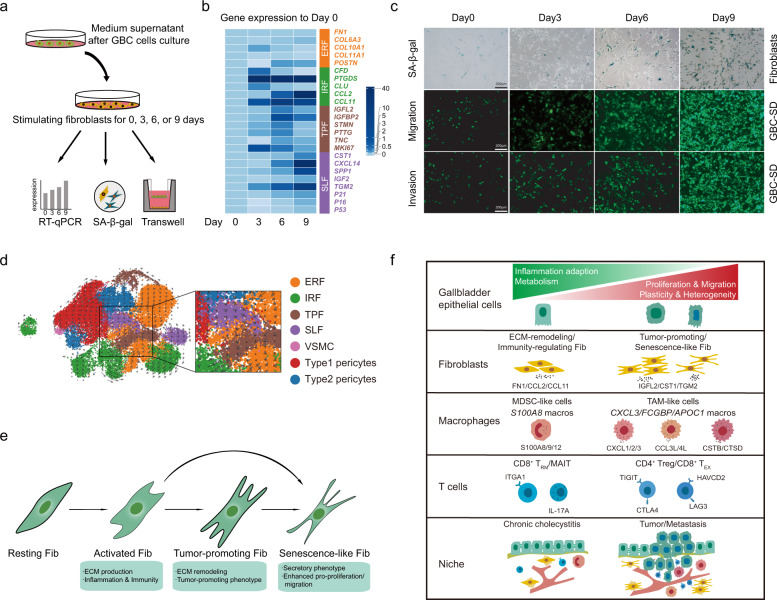
Dynamic functional states of fibroblasts and dynamics of cellular heterogeneity from inflammation to cancer. **a** Workflow displaying in vitro co-culture of human fetal lung fibroblasts (HFL-1) with medium supernatant of GBC-SD cells for 0, 3, 6, and 9 days, respectively. **b** Heatmap showing expressions dynamics of phenotypic gene signatures in HFL-1 cells when treated with medium supernatant of GBC cells, based on quantitative PCR analysis. **c** Dynamic illustration of senescence-associated β-galactosidase (SA-β-gal) staining of fibroblasts (first row), transwell cell migration assay of GBC-SD cells co-cultured with stimulated HFL-1 cells (second row), and transwell cell invasion assay of GBC-SD cells co-cultured with stimulated HFL-1 cells (third row) at 0, 3, 6, and 9 days, respectively. Scale bars, 200 μm. **d** UMAP visualization of the developmental trajectory of mesenchymal cellular subsets inferred by RNA velocity. **e** Diagram showing functional state transitions of different fibroblasts subtypes. **f** Schematic illustration overviewing dynamics of cellular heterogeneity from CC to GBC.

Multiplex immunofluorescence staining from the same patient showed enrichment of FN1^+^ (fibroblast marker) TGM2^+^ (aging marker) senescence-like fibroblasts across primary and metastatic lesions in comparison to the peritumoral tissue (Fig. 7h)^51^. When co-culturing normal human fibroblasts (HFL-1) with supernatant fluid from GBC-SD cells in vitro (Fig. 8a), HFL-1 cells showed boosted cell proliferation (*MKI67*^high^) and quiescent secretion (e.g., *CXCL14*^low^*TGM2*^low^) at day 3 (Fig. 8b). Markers for IRF (e.g., *CFD*, *CLU*, *CCL12*) and TRF (e.g., *IGFL2*, *IGFBP2*, *PTTG*) were significantly upregulated after co-culture for 3 or 6 days. By contrast, upregulation of senescence markers (*P16*, *P21*, and *P53*) and overactive secretion (e.g., *CXCL14*, *IGF2*, *TGM2*, *SPP1*), along with dampened cell proliferation (*MKI67*^low^), were noted at day 9 (Fig. 8b). Moreover, as time went by, HFL-1 cells exhibited stepwise enhanced expression of the senescence-associated β-galactosidase (SA-β-gal) and boosted pro-migration/invasion capacity, indicating the vital role of SLF in promoting GBC metastasis (Fig. 8c).

We next interrogated potential cellular differentiation trajectories of fibroblasts by RNA velocity. A cellular hierarchy was unveiled with two main trajectories: one cellular group differentiated through ERF/IRF towards TPF and then SLF; the second path went along ERF and proceeded directly towards SLF (Fig. 8d). Accordingly, we proposed a progression model for the functional state transition across fibroblast subsets (Fig. 8e).

We further investigated the interplay between fibroblasts and mEPCs by cell–cell communication analysis. ERF principally interacted with mEPCs via collagen family members and collagen receptor integrins α1β1/α2β1 (Supplementary Fig. S14). In contrast, IRF actively communicated with mEPCs through HLA-C, perhaps implying their capacity in antigen presentation (Supplementary Fig. S15). Besides, MIF-EGFR/TNFRSF14, IGF1/2-IGF1R/2 R, TNFSF12-TNFRSF12A (TWEAK-Fn14) interactions also mediated the crosstalk between TPF or SLF with EPCs, consistent with their function in facilitating tumor progression (Supplementary Figs. S16, S17 and Table S6).

Lastly, four subsets (Per01–Per04) of pericytes were identified (Supplementary Fig. S13c–e). Except for Per01, the other three subsets chiefly originated from tumor samples. Per02 and Per04 were involved in versatile tumor-promoting programs, such as blood vessel remodeling, cell adhesion, and mesenchymal stem cell properties. By comparison, Per01 and Per03 likely acted as tissue-resident pericytes, engaged in leukocyte migration, immune response, and ion transport. Altogether, these results indicated extensive functional heterogeneity and phenotypic plasticity of various stromal cells within TME, which play crucial roles in GBC progression.

## Discussion

This study deciphered components and phenotypes across cholecystitis and GBC ecosystems by scRNA-seq analysis of 140,870 cells (Fig. 8f). We identified three subtypes of cancer cells closely linked to TME cellular predilection as well as patient prognosis. We also dissected potential interactions between cancer cells and TME cells. This comprehensive single-cell transcriptome atlas provides a valuable resource for understanding mechanisms underlying the malignant transformation of chronic cholecystitis and GBC progression, further paving ways for developing precision therapies for GBC.

By comparing malignant with non-malignant epithelium, we corroborated most cancer hallmark items and, meanwhile, identified a plethora of non-canonical signatures pertinent to chromatin remodeling, mRNA translation, and protein processing, which resembled the functional partitioning of new candidate cancer genes in large-scale lung cancer or pan-cancer genomic analysis^52,53^, and implied epigenomic and translational hallmarks of GBC^18,54,55^. Meanwhile, cancer cells showed missing roles of natural mucosal guardians across epithelial integrity, mucin secretion, and transepithelial transport. Conversely, they exhibited robust differentiation, metaplastic, and stem cell-like programs, implying perturbed homeostasis, disrupted tissue organization, and development gone awry amongst GBCs^56,57^. Given the lengthy journey of preceding cholecystitis, GBC typified the essential property of CIACs under persistent cytotoxic or genotoxic stresses^4,5^.

We recapitulated two major continuum states during inflammation-driven epithelial carcinogenesis: (1) genomic stable stage (CNV^–/low^) with inflammatory adaptation^58^; (2) genomic unstable (CNV^high^) stage with inflammatory reprogramming^7^, as epitomized by the convergence of epithelial plasticity between inflamed and malignant gallbladder epithelium. Two PBM-associated benign EPC subsets (nEPC05–06), awash with regurgitated pancreatic juice, exhibited gastrointestinal metaplastic signatures. Cluster nEPC05 largely arose from tumor samples, likely implying a pre-malignant stage with not yet developed genomic instability. Another two XGC-associated clusters displayed EMP signatures, which accounted for the associated gallbladder wall destruction and inflammatory liver invasion (CC6). Unexpectedly, we captured genomic instability in a small fraction of epithelial cells from the histologically inflamed gallbladder (CC1), implying that CNV potentially occurred early and heralded morphologically malignant transformation, mirrored by previous pan-cancer evidence^59^, and meanwhile suggesting fluid boundaries between chronic inflammation and tumorigenesis stages, presumably with a transitional zone of field cancerization^60^. Of note, based on IHC validation and experimental exploration, we corroborated that elevated expression of PLA2G2A was associated with GBC initiation, corresponding with the notion that PLA2G2A may display an initial beneficial effect against gastrointestinal mucosal inflammation whereas face the tradeoff of increased cancer risk^22^. In such a way, PLA2G2A potentially wires long-term chronic cholecystitis up to GBC.

Cancer cells diverged from inflamed epithelium by cell-autonomous transcriptional switches (e.g., *NR1H4*^−^*MYC*^+^) and by chromosomal instability (e.g., chr19 segmental aberrations), whereas retaining the memory of epithelial plasticity, suggestive of sustaining co-option of ‘wound healing’ programs akin to generalized CIACs^61^. The metaplastic subgroup (subtype I) peculiarly showed KRAS-related pathway activation, together with *KRAS*^G12D^-induced tumorigenesis in our metaplastic gallbladder organoid, implying the necessity of genomic aberrations for metaplasia-induced carcinogenesis, presumably via abnormal chromatin remodeling^62^. In comparison to the ordinarily slowly self-renewal gallbladder stem cells (once every ~625 days)^63^, inflammation spawns gallbladder transit-amplifying stem cells^64^, possibly further reprogrammed into tumor-initiating stem cells by persistent inflammatory signals (e.g., NF-κB as a master)^65^, which have been pervasively identified in subtype II mEPCs. These inflammatory responses likely introduced extensive DNA damages and high mutation burdens, which consequently spurred tumor initiation, fueled tumor-elicited inflammation, and accelerated tumor progression^5,7^. We noted extensive low-grade differentiation and positive lymph nodes across subtype II patients, reminiscent of cancer-related dedifferentiation ‘hallmark’ and aberrant lymphatic vessel proliferation, both presumably orchestrated by inflammatory signals^7,18,66^. Subtype III cancer cells, typified by hybrid EMP, mostly came from metastatic lesions, wherein mesenchymal markers might confer tumor cells of survival advantage, for instance, to withstand hemodynamic fluid shear stress during cell migration.

By dissecting compartments in the gallbladder microenvironment at a single-cell resolution, we illustrated their indispensability in driving cancer hallmarks. Acting as a wound that never heals^23^, GBC at least hijacked two categories of building blocks from cholecystitis to construct its TME, pertinent to inflammation resolution (e.g., Treg cells, MDSC-like macrophages, and immune-regulating fibroblasts) and reparative ECM remolding (e.g., collagen-producing fibroblasts and macrophages), respectively. By persistently releasing inflammatory mediators and shaping a dense barrier to exclude T cell infiltration^67^, the inflammation-adapted microenvironment was gradually rewired into an immunosuppressive TME. For instance, we noted two early GBC samples enriched with *TREM2*^+^ macrophages. While aiding mucosal wound healing and restraining macrophage activation, *TREM2* might help shape an immunosuppressive TME at an early stage. Apart from these co-opted elements, GBC-TME possessed newly developed pro-tumorigenic programs, with the generic constituents mainly grouped into four classes: (1) proliferative class, typified by various *MKI67*^+^ cell subsets (e.g., fibroblasts, macrophages), conceivably related to sustaining mitogenic stimulation, genomic instability per se, and activated stem cell niches^68^; (2) pro-angiogenic class (e.g., perivascular pericytes and TAMs), presumably relevant to hypoxic and acidic TME, particularly awful when mucosal disruption resulting in stark exposure to the acidic gallbladder bile; (3) metabolic rewiring class (e.g., lipid-laden Macro01 subset), likely accounted for by oncogenic instruction (e.g., *KRAS*)^69^ or tumor-related oxidative stress^70^; (4) immunosuppressive class (e.g., tumor-promoting/senescence-like fibroblasts), possibly fueled by persistent chromosomal instability^71^ or tumor-elicited inflammation^5^.

Notwithstanding shared inflammation origins, GBC-TMEs were not created equally, as shown by *forte* and *mezzo-forte* immunosuppressive subtypes, borrowing the vivid concept in music theory^72^. The *forte* subtype corresponded to inflammatory cancer cells (subtype II) and exhibited extensive infiltration of suppressive immune cells (e.g., pDCs, M2-like macrophages, Tregs, T_EX_ cells, and polarized immune-active fibroblasts). In accordance, compared with other mEPC subtypes, subtype II mEPCs displayed more robust crosstalk with innate or adaptive immune cells. The *mezzo-forte* subtype matched well with metaplastic cancer cells (subtype I), less hijacked by suppressive cells as in the *forte* subtype, and exploited alternative tumor-promoting TME niches, exemplified by *TREM2*^+^ macrophages and tumor-promoting fibroblasts. Expanding the concept of ‘epimmunome’ in inflammatory diseases^73^, we demonstrated that the dysregulation of cancer cells could dictate the activity and plasticity of stromal and immune cell repertoires.

The high similarity between paired primary and metastatic EPC transcriptomes likely underpinned the parsimonious metastatic model of collective migration, particularly in treatment-naive GBC cases^74^, as well as the self-organized ‘histostasis’ cancer theory^57^. However, we found a handful of metastatic cancer cells exhibiting phenotypic nuance such as brisk cellular proliferation, protein synthesis, and mRNA translation, implying a central role of translational reprogramming rather than genomic changes for the adaptive cancer cell plasticity during metastatic colonization^75^, conceivably fine-tuned by the selective pressure within the metastatic niches. In line with metastatic EPCs, the stromal or immune populations in metastatic niches also displayed an increased pliancy. For instance, we noted enriched senescence-like fibroblasts in metastatic lesions, wherein copious inflammatory, extracellular modifying, and growth factors were secreted, further promoting metastasis in extensive ways (e.g., angiogenesis, EMP, and pre-metastatic niche formation)^76^. Our findings added fresh evidence to the novel cancer hallmark of cellular senescence^18^. Apart from organ adaptation, metastatic TME likely harbored distinct immunoediting programs. Firstly, we noted markedly dampened T-cell killing abilities, typified by decreased CD8^+^ T_EFF_ cytotoxicity and increased CD4^+^ Treg scores, in parallel with the culprit role of Treg cells in stimulating metastasis in mouse models^77^. Secondly, the abundance of NK cells in metastatic niches likely suggested the revitalized innate immune system to tackle poorly antigenic cancer cells that escaped T cell immunosurveillance^78^. Thirdly, metastatic lesions represented primary sources of IFN-associated macrophages, reminiscent of upregulated ISGs in the pre-malignant epithelium. The paradoxical bimodal peak of IFN signals implied its co-option during tumor metastasis^79^, possibly involved in adaptive immune resistance of cancer cells and chromosomal instability-related cytosolic DNA responses, which might be conservative across human cancers^80^.

Inflammation-driven carcinogenesis has been chiefly informed by in vitro studies or genetic animal models. At an in vivo single-cell level and across a continuum of disease states, we systematically elucidated how the gallbladder cellular fuels burn up the smoldering inflammatory fire into raging cancer fire, either within cancer cells or from their goldilocks zone. GBC subverts long-term inflammatory tissue homeostasis and establishes its own hierarchy by usurping extensive ‘wound healing’-like programs, seeking new pro-tumorigenic players, rewiring new crosstalk circuits and, more strikingly, re-organizing them together into seemingly perpetual and indestructible machinery. Herein, many cellular elements play as delicate rheostats rather than static building blocks, exhibiting dynamic and context-dependent phenotypic or functional plasticity, which underscores the GBC heterogeneity and explains the universal frustration in ‘targeting’ GBC. Furthermore, the metaplastic, inflammatory, and mesenchymal classification system likely expands our knowledge regarding precision chemo-and immunotherapy of GBC. Implied by cancer cell and TME signatures, it is reasonable to presume that inflammatory subtype II GBCs were more suitable for immune checkpoint blockade therapy. Comparatively, the other two subtypes might be better managed by incorporating stromal-normalization therapies. It is worthwhile to uncover whether the GBC chemotherapy regimens could, analogous to ampullary carcinoma (pancreaticobiliary versus intestinal subtype)^81^, be instructed by the gastrointestinal metaplastic state. In addition, targeting the senescence-like fibroblasts in the tumor stroma might be a promising strategy to retard GBC metastasis.

In conclusion, the comparative profiling of inflamed gallbladders, primary GBCs, and matched metastases delineated the molecular features that drive GBC development and progression, laying a new foundation for future hypothesis-driven translational research. Further large-scale multi-omics datasets contributed by multicenter collaboration are expected to verify our scRNA-seq findings and elucidate the immunosuppressive or treatment-resistant enigmas underlying this intractable disease.

## Materials and methods

### Human specimens

Patient-derived samples for this study were collected at Eastern Hepatobiliary Surgery Hospital (EHBH), Shanghai, China. Details for patients in the scRNA-seq cohort were shown in Supplementary Table S1. The tissue microarray cohort included 49 GBC patients who underwent curative intended resection at EHBH from May 2017 to Mar 2019 (Supplementary Table S8). All patients’ diagnoses were histologically confirmed. This cohort was followed up until June 30th 2020, with a median follow-up of 17.7 months (range, 0.5–32.2 months). Variables collected included gender, age, anti-HBc status, gallstone, gallbladder polyps, CEA level, CA 19-9 level, location, type of surgery, surgical margin, liver invasion, vascular invasion, bile duct invasion, tumor stage, lymph node metastasis, distant metastasis, TNM stage, tumor differentiation, and histological type. This study was approved by the Ethical Committee of EHBH (2018-1-001) and was conducted in accordance with the Declaration of Helsinki. The informed consent was signed by each participant.

### Single-cell isolation and sequencing

Tissues were cut into about 0.4 × 0.4 mm and transported in tissue storage solution, and then washed with DPBS before being minced. To get single-cell suspensions, the samples were processed as follows: minced; dissociated with digestant (0.25% trypsin (ThermoFisher)) and 10 μg/mL DNase I (Sigma) dissolved in PBS with 5% FBS (ThermoFisher) and incubated in 37 °C with a shaking speed of 50 rpm for about 40 min; repeatedly collected dissociated cells at an interval of 20 min; filtered by a 40 μm nylon cell strainer; removed red blood cells by 1× Red Blood Cell Lysis Solution (ThermoFisher); washed with DPBS containing 2% FBS. The viability of cells would be checked on Countess^®^ II Automated Cell Counter (ThermoFisher) after trypan blue staining (ThermoFisher).

To ensure each cell paired with a bead in a Gel Beads-in emulsion (GEM), 10× library preparation and sequencing beads with the unique molecular identifier (UMI) and cell barcodes were loaded close to saturation. Polyadenylated RNA molecules hybridized to the beads after exposure to the cell lysis buffer. Beads were retrieved into a single tube for reverse transcription. On cDNA synthesis, each cDNA molecule was tagged on the 5’ end with UMI and cell label indicating its cell of origin. Briefly, 10× beads were then subject to second-strand cDNA synthesis, adaptor ligation, and universal amplification. Sequencing libraries were prepared using randomly interrupted whole-transcriptome amplification products to enrich the 3’ end of the transcripts linked with the cell barcode and UMI. All the remaining procedures including the library construction were performed according to the standard manufacturer’s protocol (CG000206 RevD). Sequencing libraries were quantified using a High Sensitivity DNA Chip (Agilent) on a Bioanalyzer 2100 and the Qubit High Sensitivity DNA Assay (ThermoFisher). The libraries were sequenced on NovaSeq6000 (Illumina) using 2 × 150 chemistry (Shanghai Bohao Biotechnology; Shanghai OEbiotech Corporation).

### DNA isolation and whole exon sequencing (WES)

Genomic DNA of samples was extracted by QIAamp DNA Mini Kit (Qiagen) and then captured with an Agilent SureSelect Human All Exon Kit V6 (Agilent). DNA libraries were generated following the protocols provided by Illumina. DNA libraries were sequenced with the Illumina NovaSeq 6000 System (Illumina), yielding 150 bp of paired-end sequence, and FASTQ files were generated. The WES sequencing and analysis were conducted by OE Biotech Co., Ltd. (Shanghai, China).

### Single-cell RNA-seq data processing

Raw gene expression matrices were generated for each sample by the Cell Ranger Pipeline coupled with human reference version GRCh38. The output filtered gene expression matrices were analyzed by R software with the Seurat package^82^. In brief, genes expressed at a proportion > 0.1% of the data and cells with >200 genes detected were selected for further analyses. Low-quality cells were removed if they met the following criteria: (1) <200 genes; (2) >20% UMIs derived from the mitochondrial genome. After removal of low-quality cells, the gene expression matrices were normalized by the NormalizeData function, and 2000 features with high cell-to-cell variation were calculated using the FindVariableFeatures function. To reduce the dimensionality of the datasets, the RunPCA function was conducted with default parameters on linear-transformation scaled data generated by the ScaleData function. Next, the ElbowPlot, DimHeatmap functions were used to identify the proper dimension of each dataset. Finally, we clustered cells using the FindNeighbors (top 22 PCs) and FindClusters functions (resolution = 0.8), and performed non-linear dimensional reduction with the RunTSNE function with default settings.

Based on the above analysis, we separated and clustered each cell type with the same process. For mesenchymal cells, we selected the top 15 PCs and resolution at 0.3. For T cells, we select the top 15 PCs and resolution at 1.2. For epithelial cells, we selected the top 20 PCs and resolution at 0.2. For macrophages, we selected the top 14 PCs and resolution at 0.15. For endothelial cells, we selected the top 14 PCs and resolution = 0.1.

### Cell type annotation and cluster markers identification

After non-linear dimensional reduction and projection of all cells into two-dimensional spaces by *t-*SNE, cells clustered together according to common features. The FindAllMarkers function with default parameters in Seurat was used to find markers for each of the identified clusters. Clusters were then classified and annotated based on expression of canonical markers of particular cell types.

### DEGs identification and functional enrichment

Differential gene expression testing was performed using the FindMarkers function in Seurat with parameter “test.use = wilcox”, only.pos = TRUE, and other parameters by default, and the Benjamini-Hochberg method was used to estimate the adjusted *P* value. DEGs were filtered using a minimum log (fold change) of 0.25 and a maximum adjusted *P* value of 0.01. Enrichment analysis for the functions of the DEGs was conducted using clusterProfiler R package^83^. Gene set variation analysis (GSVA) was performed using gene sets obtained from the C6 molecular signature database using default sets, as described in the GSVA package^84^. To depict the function preference of each cell cluster, we calculated the preferential expression of signature genes. The potential roles of GBC-related DEGs were mapped by using the Cancer Hallmarks Analytics Tool (http://chat.lionproject.net/)^85^, signature gene sets in GSEA database (http://www.gsea-msigdb.org/), or the Human Protein Atlas (https://www.proteinatlas.org/).

### CNV analysis

To identify malignant cells with clonal large-scale chromosomal CNVs, we used the inferCNV R package^86^ to infer the genetic profiles of each cell based on the average expression of large gene sets (101 genes) in each chromosomal region of the tumor genome compared to normal cells. All epithelial cells and 240 endothelial cells (10 cells of each sample) were input as interrogation group, and 100 endothelial cells from each patient were sampled randomly as control. Other parameters were set as default.

### SCENIC analysis

The SCENIC analysis was run using the R package SCENIC^87^. The co-expression modules were run by GENIE3 (SCENIC 1.1.2-2). The motifs database for Homo sapiens was downloaded from the website https://resources.aertslab.org/cistarget/databases/. The input matrix was the normalized expression matrix of cells of interest.

### Cell-cell communication analysis

We used CellPhoneDB for data analysis^88^. Ligand-receptor pairs with *P* values > 0.05 were filtered, while the others were retained for evaluating the relationship between the different cell types.

### RNA velocity

RNA velocity was performed to investigate the potential inter-relationship of mesenchymal cell lineage using velocyto^89^. BAM file containing all the mesenchymal cells was used in this pipeline, spliced/unspliced reads were annotated. The calculation of RNA velocity values for each gene in each cell and embedding RNA velocity vector to low-dimension space was done by following the velocyto pipeline. All the parameters were set as default. The result was visualized into UMAP plot.

### Tissue distribution preference of clusters

To define the tissue preference of each cell cluster, we calculated the ratio of observed to excepted cell numbers (Ro/e) of each cluster in different tissues^90^. The expected cell numbers for each combination of cell clusters and tissues are obtained from the chi-square test. One cluster was enriched in a specific tissue if Ro/e > 1. Heatmaps were generated by pheatmap package.

### Functional analysis for T cells

Cytotoxicity and exhaustion signatures were derived from differentially expressed genes across all CD8^+^ T cell subtypes. Pearson correlation between the reference gene *GZMK* (cytotoxicity signature) or *HAVCR2* (exhaustion signature) and all other genes across CD8^+^ T cells using scaled expression values was analyzed. The top 30 genes having the highest correlation with the reference genes (*GZMK* or *HAVCR2*) were defined as cytotoxicity and exhaustion signature genes, respectively. For CD4^+^ T cells, the *IL2RA* gene was chosen as the reference gene for defining the Treg signature using the same method. We computed signature scores for individual cells using AddModuleScore function in Seurat.

### WES analysis

The raw reads in fastq format were pre-processed with fastp^91^. Firstly, Adapter sequences and sequences with an average quality value below 15 bases in a sliding window were trimmed. Then, clean reads were aligned to the reference human genome GRCh37 using the BWA^92^. The mapped reads were sorted and indexed by SAMtools^93^, following Picard (Version 4.1.0.0) for marking duplicate reads, to obtain analysis-ready BAM files which were used as input files for variant calling. The GATK (Version 4.1.9.0)^94^ was used for recalibration of the base quality score and single nucleotide polymorphism (SNP) and insertion/deletion (INDEL) realignment. The annotation databases, such as Refseq, 1000 Genomes, the Catalogue of Somatic Mutations in Cancer (COSMIC), OMIM, EXAC, esp6500, gnomAD, SIFT, clinvar, PolyPhen, MutationTaster, gwasCatalog, and OMIM were referred to during SNP&INDEL calling and annotated using ANNOVAR^95^. Specifically, copy number variation (CNV) was inferred from sequencing data using the software package CNVkit (version 0.9.8)^96^, and Lumpy software (Version 0.2.13)^97^ was applied to call structural variation (SV). Tumor mutation burden (TMB) was defined as the number of somatic, coding, base substitution, and indel mutations per megabase of genome examined. In this study, we calculated TMB as the number of all nonsynonymous mutations/exon length for each sample.

### Validation cohort

We acquired the bulk RNA-seq data of 111 GBC patients from the European Genome-phenome Archive (EGA) with permission. The fastq files were processed using Trimmomatic with low-quality reads removed^98^. The clean reads were mapped to the human genome (GRCh38) using HISAT2^99^. The read counts of each gene were obtained by HTSeq-count^100^. To validate the subtypes based on the malignant epithelium in our cohort, we used the DESeq2 function ‘rlog’ to regularize log transform of the normalized count values^101,102^. Then, the characteristic gene sets of each subtype were constructed with the top 15 genes of themselves, and gene set variation analysis (GSVA) was performed by GSVA packages with default parameters. Finally, the patients were classified by hierarchical clustering with hclust function in R and the heatmap was generated by Complexheatmap package^103^.

To verify the relationship between malignant epithelial subtypes and tumor microenvironment, we used the TIMER2 and CIBERSORT to predict the infiltration of immune cells^104,105^. Also, we calculated the correlation of the top 15 genes of each subtype and T subcluster signatures. Heatmaps were generated by corrplot package.

### IHC analysis

The IHC was performed as previously described^106^. To measure the immunoreactivity of different markers, the pathologists who as blinded to patients’ outcomes performed the image analysis based on the staining of density and intensity. The scores were as follows: 1 for 0%–25% density or negative intensity, 2 for 26%–50% density or weak intensity, 3 for 51%–75% density or medium intensity, and 4 for 76%–100% intensity or strong intensity. The final semi-quantitative score was acquired by the density score multiplying the intensity score, ranging from 1 to 16.

### Multiplex immunofluorescence staining

The procedure of deparaffinization, rehydration, and endogenous peroxidases quench was conducted as standard IHC. In general, all incubation was conducted in a dark moisture chamber and at 300 rpm on an orbital shaker. Applied liquids were removed and washed three times with TBST after each step. Then one cycle of the multi-color staining protocol was performed according to the instructions (PerkinElmer). Images of immunofluorescence stained slides were acquired from the co-focal microscope using the 60× objective with saturation protection as a whole-slide overview. A spectral library was constructed with each OPAL-fluorochrome. The same spectral library was used for all analyzed immunofluorescence panels throughout the experiments.

### Tumor cell viability, apoptosis, and stemness assays

Tumor cell viability was measured by the CCK8 assay. Cells were seeded in 96-well plates at a density of 2000 cells per well. Cells were incubated with CCK8 solution (1:10 v/v) for 2 h every 24 h. The absorbance was measured at 450 nm by microplate reader. Cell apoptosis was detected by using the TUNEL Assay Kit in accordance with the manual. We measured the expression of stem cell markers (CD44, EpCAM) using flow cytometry analysis. In brief, cells were collected and resuspended in 100 µL PBS (1% BSA and 0.1% sodium azide) and were stained with APC-EpCAM or PE-CD44 antibodies for 30 min. The results were analyzed by FlowJo V10. In sphere formation assays, 1000 cells per well were seeded in the ultra-low attachment 6-well plates. Tumor spheres larger than 75 μm were counted under stereomicroscope after 10 days.

### Gallbladder organoid (GBO) establishment and assessment

Fresh gallbladder tissues were obtained with informed consent from patients who underwent surgery at EHBH. Briefly, the tumor tissues were washed with PBS for 1–2 times, minced into 1 mm^3^ with scissors, and incubated at 37 °C in digestion solution (Dulbecco’s Modified Eagle Medium (DMEM)) with 4 mg/mL collagenase D (Roche), 10 μM Y27632 (Sigma-Aldrich), and 1× Primocin (InvivoGen) on an orbital shaker for 1–2 h until no visible cell mass could be seen. Then, digestion was stopped by adding the cold termination medium (DMEM medium with 1% penicillin/streptomycin, 1× primocin, 10 μM Y27632, 10% FBS). The cell suspension was filtered through a 70 μm Nylon cell strainer and washed with cold Advanced DMEM/F12 twice before spinning at 300–400× *g* for 5 min. Re-suspension of the cells in cold human liver organoid medium mixed with Matrigel and was seeded into a 6/24-multiwell plate at 37 °C for 1 h. After polymerization of matrix, the human liver organoid medium was added to each well. The culture was generally changed every 3–4 days. After 1–2 weeks, the organoids were repeatedly blown with a gun tip to disperse the cells and then replanted into Matrigel at a ratio of 1: 2.

The GBOs were fixed in 10% formalin and embedded in paraffin. The paraffin-embedded sections (4 µm thick) were prepared and stained with H&E using standard protocols. IHC staining was performed as described above.

### GBO and GBC cell line lentivirus infection

The green fluorescent protein (*GFP*), *PLA2G2A* or *KRAS*^G12D^ CDS sequence was inserted into pLenti-CMV-3FLAG lentiviral vector (OBiO Technology, Shanghai). GBC cell lines (GBC-SD and NOZ) were purchased from Cell Bank of Type Culture Collection of Chinese Academy of Sciences. GBC-SD and NOZ cells were transfected with lentivirus of NC labeling GFP or *PLA2G2A* labeling GFP with a multiplicity of infection (MOI) 20 for 4 h. After 12 h, the original medium was replaced with fresh medium. Then the cells were selected with puromycin for two weeks before performing migration and invasion experiments.

In terms of GBO lentivirus infection, the organoids were resuspended in a 500 μL growth medium after trypsinization for 5 min at 37 °C. Then cells were seeded into 48-well plates at 80%–90% confluence and were infected with lentivirus of NC labeling GFP and *KRAS*^G12D^ labeling GFP according to standard procedures^107^. Three days after infection, the growth medium was exchanged with medium containing puromycin at a concentration of 5 μg/mL for selection for two weeks.

### Co-culture experiment and senescence-associated β-galactosidase (SA-β-gal) assay

Human fetal lung fibroblasts (HFL-1) were purchased from Cell Bank of Type Culture Collection of Chinese Academy of Sciences. For cell co-culture, the medium supernatant collected from GBC-SD cells was used to culture HFL-1 for 0, 3, 6, and 9 days, respectively. Then the senescence β-Galactosidase Staining Kit was used to detect the senescence of HFL-1 according to the manufacturer’s instructions. In brief, cells were fixed 4% paraformaldehyde for 15 min at room temperature before being incubated with SA-β-gal staining solution overnight at 37 °C without CO_2_. The stain of SA-β-gal was visualized under a Zeiss microscope.

### Total RNA extraction and quantitative real-time PCR (qRT-PCR)

The procedures of RNA extraction and qPCR were performed as previously described. Primers used for qRT-PCR are listed in Supplementary Table S14. Expression levels were calculated using the 2^−ΔΔCT^ method with β-actin as the control.

### Invasion and migration assays

Both invasion and migration assays were conducted by using 8.0 mm Boyden chambers (BD Biosciences). For invasion assay, the Boyden chambers were covered with 200 μL of phenol-red-free matrigel mix which was diluted 1:40 portions with DMEM. Thereafter, these chambers were placed in a 24-well plate and were incubated for 20 mins at 37 °C. For invasion and migration assays of GBC cells, the wells of the lower chamber were filled with medium containing 10% FBS. GBC cells (2 × 10^4^) were seeded in the upper compartment in serum-free medium for 24 h. At the end of assay, filters were removed and fixed. The invasion and migration were determined by counting the cells that migrated to the lower side of the filter. For invasion and migration assays in co-culture system, GBC-SD cells (2 × 10^4^) labeling GFP were platted under the serum-starved condition in the upper chambers, while in the lower chambers, HFL-1 cells (2 × 10^4^) that co-cultured with medium supernatant of GBC-SD cells for 0, 3, 6, and 9 days were seeded. The experiment was stopped after 24 h of incubation. An equal number of cells were seeded in wells underneath to normalize the invasion and migration assay to cell proliferation.

### Primers and antibodies

The primers, primary and secondary antibodies were listed in Supplementary Table S14, and were used at the concentrations indicated by manufacturer’s instructions.

### Statistics

Based on the median IHC scores of markers, the GBC specimens were divided into high and low groups. The R package ‘surviminer’ and ‘survival’ were used for Kaplan-Meier survival analysis. Details of statistical tools, methods, and thresholds for statistical analysis are described in the respective results section, methods, and figure legends.

## Supplementary information

Supplementary Figures S1-S17

Supplementary Table S1 Informations for samples in sc-RNA seq analysis

Supplementary Table S2 The number of cells in each sample

Supplementary Table S3 DEGs (nEPC vs mEPC)

Supplementary Table S4 Overview of the top100 overexpressed genes in mEPC

Supplementary Table S5 Overview of the top100 overexpressed genes in nEPC

Supplementary Table S6 Description of typical transcriptional factors, metastasis-promoting genes, and representative ligand-receptor pairs

Supplementary Table S7 Malignant epithelial cell subtypes Top100 DEGs

Supplementary Table S8 Characteristics of GBC patients in tissue microarray

Supplementary Table S9 Macrophage and DC clusters Top50 DEGs

Supplementary Table S10 Macrophages function preference markers

Supplementary Table S11 T and NK subsets Top50 DEGs

Supplementary Table S12 T cells function preference markers

Supplementary Table S13 Fibroblast subtypes Top50 DEGs

Supplementary Table S14 Key resources
