# Supplementary material for: Single-cell dissection of remodeled inflammatory ecosystem in primary and metastatic gallbladder carcinoma
Source: Cell Discov. 2022 Oct 5;8:101. doi: 10.1038/s41421-022-00445-8 (PMC9534837; doi:10.1038/s41421-022-00445-8)
Supplement: Supplementary file 1 — Supplementary Figures S1-S17 [file 41421_2022_445_MOESM1_ESM.pdf]

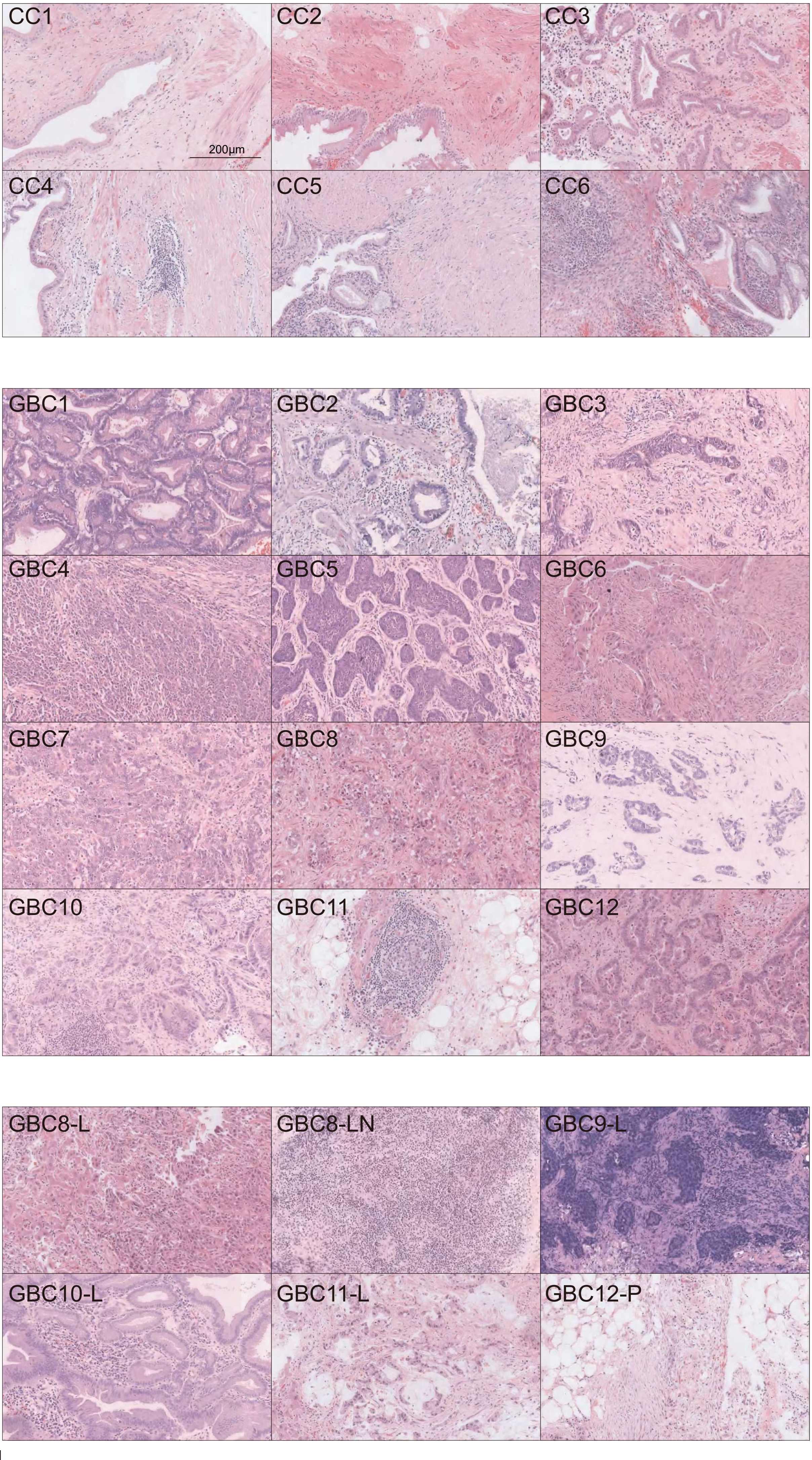
CC1
200μm
CC2
CC3
CC4
CC5
CC6
GBC1
GBC2
GBC3
GBC4
GBC5
GBC6
GBC7
GBC8
GBC9
GBC10
GBC11
GBC12
GBC8-L
GBC8-LN
GBC9-L
GBC10-L
GBC11-L
GBC12-P

Fig.S1

Supplementary Fig. S1. Hematoxylin and eosin (H&E) staining of all samples in our scRNA-seq cohort (n = 24). Scale bars, 200 µm. CC, chronic cholecystitis; GBC, gallbladder carcinoma.

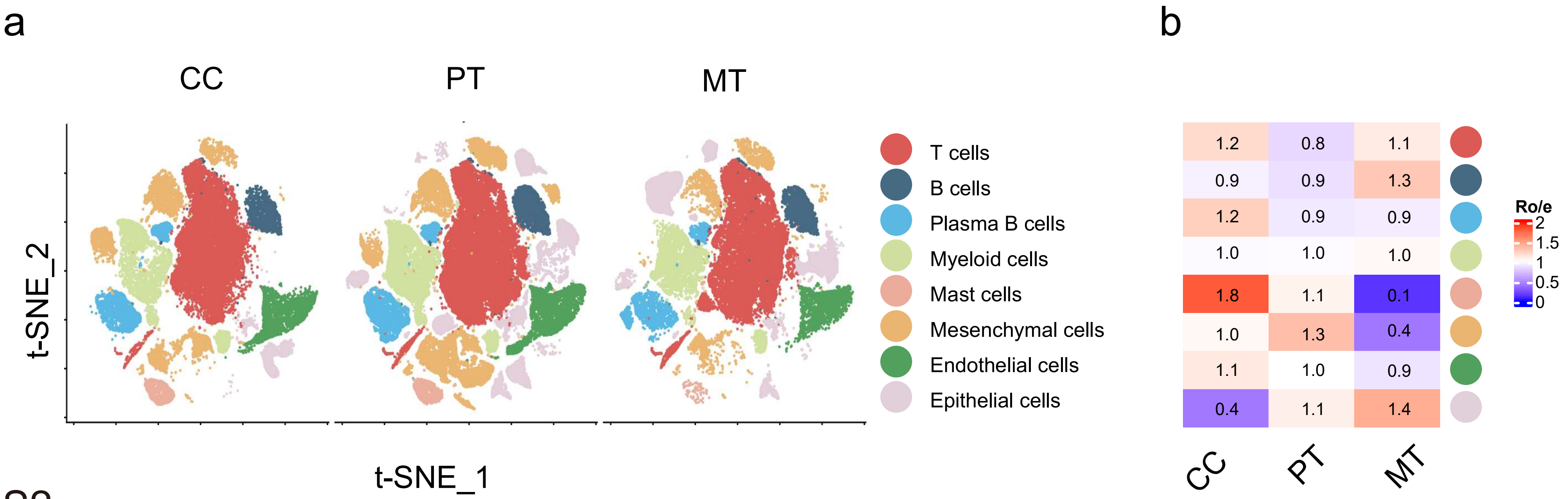
a
CC
PT
MT
t-SNE_2
t-SNE_1
T cells
B cells
Plasma B cells
Myeloid cells
Mast cells
Mesenchymal cells
Endothelial cells
Epithelial cells
b
1.2 0.8 1.1
0.9 0.9 1.3
1.2 0.9 0.9
1.0 1.0 1.0
1.8 1.1 0.1
1.0 1.3 0.4
1.1 1.0 0.9
0.4 1.1 1.4
Ro/e
2
1.5
1
0.5
0
CC
PT
MT

Fig.S2

Supplementary Fig. S2. Comparison of cellular composition between CCs, PTs, and MTs. a t-SNE plots visualizing the distribution of cell types across CCs, PTs, and MTs. **b** Heatmap showing the preferential enrichment of various cell types across CCs, PTs, and MTs. Cell types are colored in line with **a**. Ro/e > 1 indicates significant enrichment.

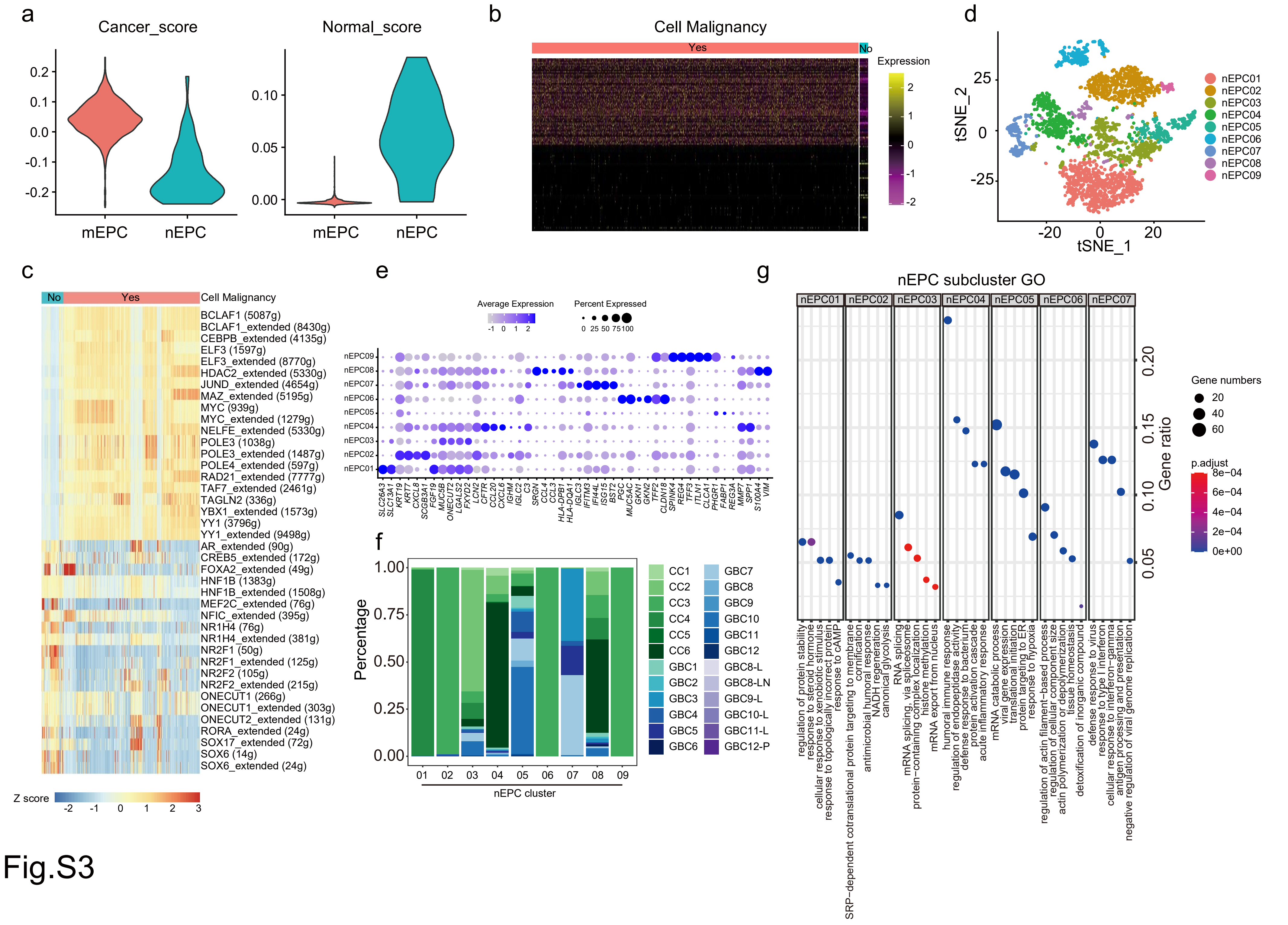
a
Cancer_score
Normal_score
mEPC
nEPC
b
Cell Malignancy
Yes
No
Expression
c
Cell Malignancy
BCLAF1 (5087g)
BCLAF1_extended (8430g)
CEBPB_extended (4135g)
ELF3 (1597g)
ELF3_extended (8770g)
HDAC2_extended (5330g)
JUND_extended (4654g)
MAZ_extended (5195g)
MYC (939g)
MYC_extended (1279g)
NELFE_extended (5330g)
POLE3 (1038g)
POLE3_extended (1487g)
POLE4_extended (597g)
RAD21_extended (7777g)
TAF7_extended (2461g)
TAGLN2 (336g)
YBX1_extended (1573g)
YY1 (3796g)
YY1_extended (9498g)
AR_extended (90g)
CREB5_extended (172g)
FOXA2_extended (49g)
HNF1B (1383g)
HNF1B_extended (1508g)
MEF2C_extended (76g)
NFIC_extended (395g)
NR1H4 (76g)
NR1H4_extended (381g)
NR2F1 (50g)
NR2F1_extended (125g)
NR2F2 (105g)
NR2F2_extended (215g)
ONECUT1 (266g)
ONECUT1_extended (303g)
ONECUT2_extended (131g)
RORA_extended (24g)
SOX17_extended (72g)
SOX6 (14g)
SOX6_extended (24g)
Z score
d
tSNE_1
tSNE_2
nEPC01
nEPC02
nEPC03
nEPC04
nEPC05
nEPC06
nEPC07
nEPC08
nEPC09
e
Average Expression
Percent Expressed
f
Percentage
nEPC cluster
CC1
CC2
CC3
CC4
CC5
CC6
GBC1
GBC2
GBC3
GBC4
GBC5
GBC6
GBC7
GBC8
GBC9
GBC10
GBC11
GBC12
GBC8-L
GBC8-LN
GBC9-L
GBC10-L
GBC11-L
GBC12-P
g
nEPC subcluster GO
Gene ratio
Gene numbers
p.adjust
regulation of protein stability
response to steroid hormone
cellular response to xenobiotic stimulus
response to topologically incorrect protein
response to cAMP
SRP-dependent cotranslational protein targeting to membrane
cornification
antimicrobial humoral response
NADH regeneration
canonical glycolysis
RNA splicing
mRNA splicing, via spliceosome
protein-containing complex localization
histone methylation
mRNA export from nucleus
humoral immune response
regulation of endopeptidase activity
defense response to bacterium
protein activation cascade
acute inflammatory response
mRNA catabolic process
viral gene expression
translational initiation
protein targeting to ER
response to hypoxia
regulation of actin filament-based process
regulation of cellular component size
actin polymerization or depolymerization
tissue homeostasis
detoxification of inorganic compound
defense response to virus
response to type I interferon
cellular response to interferon-gamma
antigen processing and presentation
negative regulation of viral genome replication

Fig.S3

Supplementary Fig. S3. Discrimination of mEPCs and nEPCs, and characterization of nPECs. a Violin plots comparing tumor gene scores and normal gene set scores between CNV-inferred mEPCs and nEPCs in GBC7, respectively. The scoring system was defined by the top 50 DEGs based on bulk RNA-seq data (paired tumoral and peritumoral tissues from GBC7). **b** Heatmap showing the top 50 DEGs identified by bulk RNA-seq analyses of GBC7 sample and its paired peritumoral sample. **c** Heatmap comparing activities of transcription factors between mEPCs and nEPCs, based on SCENIC analysis. **d** t-SNE plot visualizing color-coded nEPC clusters (n = 9). **e** Bubble plots showing expressions of phenotype-specific gene signatures per nEPC cluster. **f** Distribution of samples across different nEPC clusters. **g** Dot plots showing typically significantly enriched GO pathways among nEPC clusters.

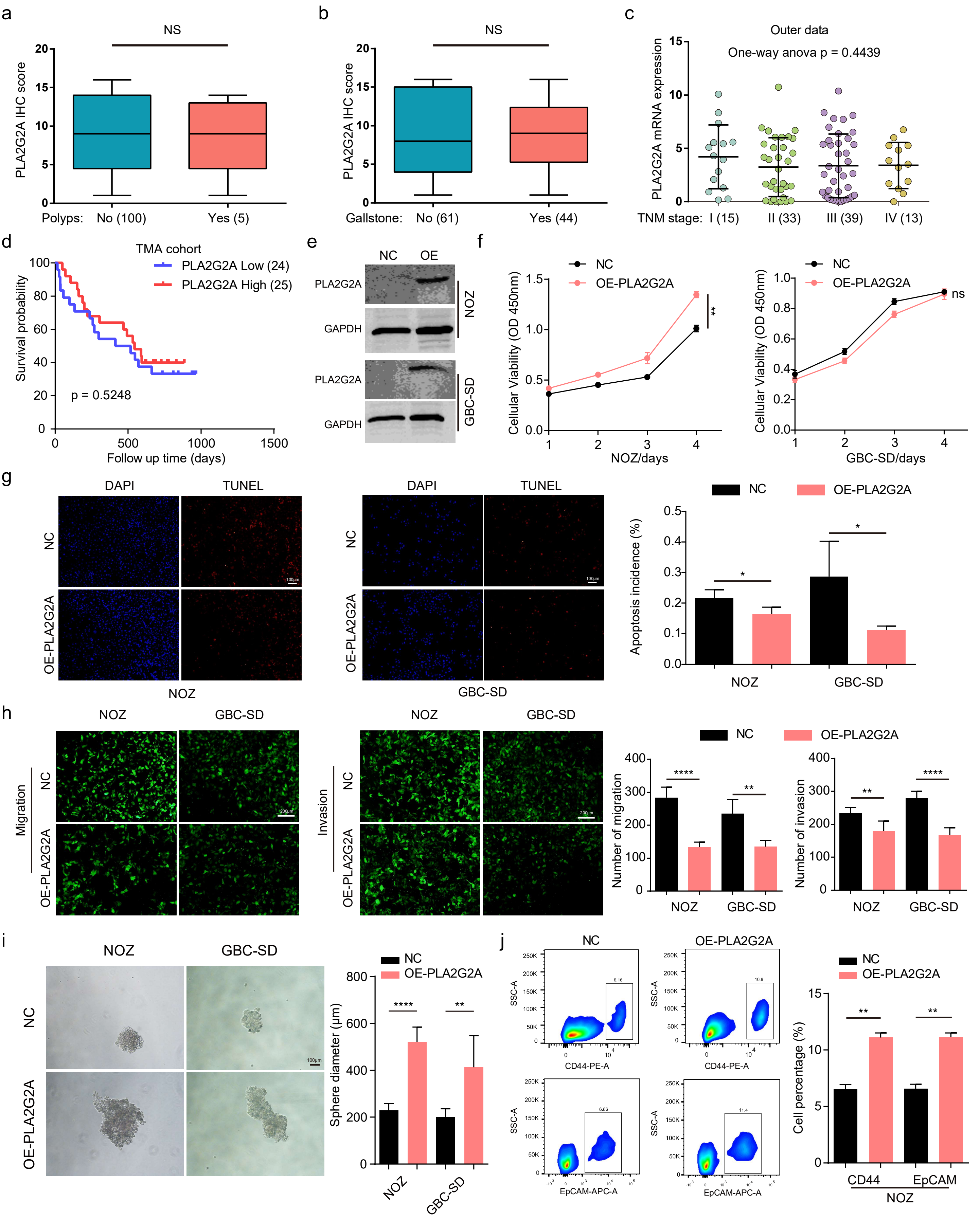

a
NS
PLA2G2A IHC score
Polyps: No (100) Yes (5)
b
NS
PLA2G2A IHC score
Gallstone: No (61) Yes (44)
c
Outer data
One-way anova p = 0.4439
PLA2G2A mRNA expression
TNM stage: I (15) II (33) III (39) IV (13)
d
TMA cohort
PLA2G2A Low (24)
PLA2G2A High (25)
Survival probability
p = 0.5248
Follow up time (days)
e
NC OE
PLA2G2A
GAPDH
NOZ
GBC-SD
f
NC
OE-PLA2G2A
Cellular Viability (OD 450nm)
NOZ/days
GBC-SD/days
ns
g
DAPI TUNEL
NC
OE-PLA2G2A
NOZ
GBC-SD
Apoptosis incidence (%)
h
Migration
Invasion
Number of migration
Number of invasion
i
Sphere diameter (μm)
j
CD44-PE-A
EpCAM-APC-A
SSC-A
Cell percentage (%)
CD44 EpCAM

Fig.S4

Supplementary Fig. S4. Association between PLA2G2A and clinico-biological features of gallbladder carcinoma. a Boxplots comparing the expression of PLA2G2A between polyps-related GBCs (n = 5) and GBCs without polyps (n = 100) based on IHC staining. Wilcoxon rank-sum test; NS, not significant. **b** Boxplots comparing the expression of PLA2G2A between gallstone-related GBCs (n = 44) and GBCs without gallstones (n = 61) based on IHC staining. Wilcoxon rank-sum test; NS, not significant. **c** Beeswarm plots displaying the transcription level of PLA2G2A across different TNM stages of GBCs based on bulk RNA-seq data from an external GBC cohort (n = 100). One-way ANOVA test; $P = 0.44$. **d** Kaplan-Meier curves of overall survival when stratifying patients by high or low expression of PLA2G2A, based on our GBC tissue microarray (n = 49). Log-rank test, $P = 0.52$. TMA, tissue microarray. **e** Western blot analysis verifying increased PLA2G2A expression in *PLA2G2A*-overexpressing GBC cell lines (NOZ, GBC-SD) as compared with control cells. NC, normal control; OE, overexpression. **f** CCK8 assays comparing cell growth capacity between *PLA2G2A*-overexpressing cells and control cells. Left panel: NOZ cells, $^{**}P < 0.01$; right panel: GBC-SD cells; ns, no significance. *P* value was calculated using two-sided unpaired Student's t test. **g** Representative images and quantification of TUNEL assays (marking apoptotic bodies) in *PLA2G2A*-overexpressing GBC cell lines versus control cells. DAPI: blue, TUNEL: red. Scale bars, 100 μm. Left panel: TUNEL staining in NOZ cells; middle panel: TUNEL staining in GBC-SD cells; right panel: boxplots of apoptosis index. Two-sided unpaired Student's t test. $^{*}P < 0.05$. **h** Representative images and quantitative boxplots showing transwell migration and invasion assays in *PLA2G2A*-overexpressing GBC cell lines (NOZ, GBC-SD) versus control cells. From left to right: migration assay, invasion assay, boxplots quantifying migration capacity, boxplots quantifying invasion capacity. Two-sided unpaired Student's t test. $^{**}P < 0.01$; $^{****}P < 0.0001$. Scale bars, 200 μm. **i** Representative images and quantification of colony formation assay in *PLA2G2A*-overexpressing GBC cell lines (NOZ, GBC-SD) versus control cells. Scale bars, 100 μm. Two-sided unpaired Student's t test. $^{**}P < 0.01$; $^{****}P < 0.0001$. **j** Representative images and quantification based on flow cytometry analysis of stem cell markers (CD44, EpCAM) in *PLA2G2A*-overexpressing GBC cell line (NOZ) versus control cells. Left: markers in control cells; middle: markers in *PLA2G2A*-overexpressing cells; right: boxplots quantifying positive cells. Two-sided unpaired Student's t test. $^{**}P < 0.01$.

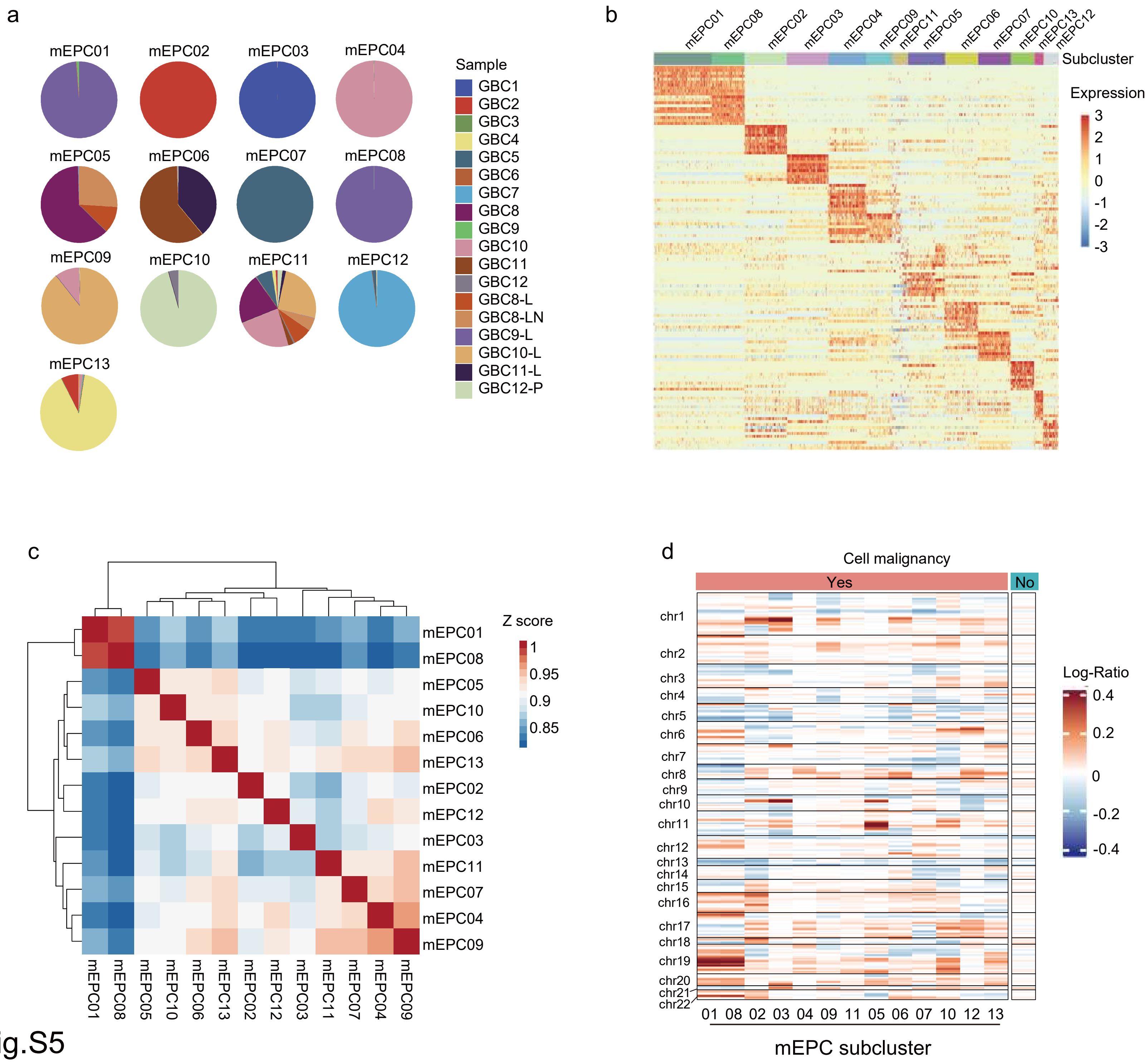
a
mEPC01
mEPC02
mEPC03
mEPC04
mEPC05
mEPC06
mEPC07
mEPC08
mEPC09
mEPC10
mEPC11
mEPC12
mEPC13
Sample
GBC1
GBC2
GBC3
GBC4
GBC5
GBC6
GBC7
GBC8
GBC9
GBC10
GBC11
GBC12
GBC8-L
GBC8-LN
GBC9-L
GBC10-L
GBC11-L
GBC12-P
b
mEPC01 mEPC08 mEPC02 mEPC03 mEPC04 mEPC09 mEPC11 mEPC05 mEPC06 mEPC07 mEPC10 mEPC13 mEPC12
Subcluster
Expression
3
2
1
0
-1
-2
-3
c
mEPC01
mEPC08
mEPC05
mEPC10
mEPC06
mEPC13
mEPC02
mEPC12
mEPC03
mEPC11
mEPC07
mEPC04
mEPC09
Z score
1
0.95
0.9
0.85
d
Cell malignancy
Yes
No
chr1
chr2
chr3
chr4
chr5
chr6
chr7
chr8
chr9
chr10
chr11
chr12
chr13
chr14
chr15
chr16
chr17
chr18
chr19
chr20
chr21
chr22
Log-Ratio
0.4
0.2
0
-0.2
-0.4
01 08 02 03 04 09 11 05 06 07 10 12 13
mEPC subcluster

Fig.S5

Supplementary Fig. S5. Comparison of transcriptional profiles between malignant epithelial clusters. **a** Pie charts showing the relative abundance of tumor samples (n = 18) across different malignant epithelial clusters (mEPC01-13). **b** Gene expression heatmap showing top-ranking DEGs for malignant epithelial clusters (n = 12). **c** Correlation heatmap displaying similarities of transcriptional profiles between mEPC subsets, color-coded by *Z* scores. **d** Heatmap showing distributions of inferred CNV hotspots (copy number gains or losses) across the chromosomal regions within different mEPC subsets.

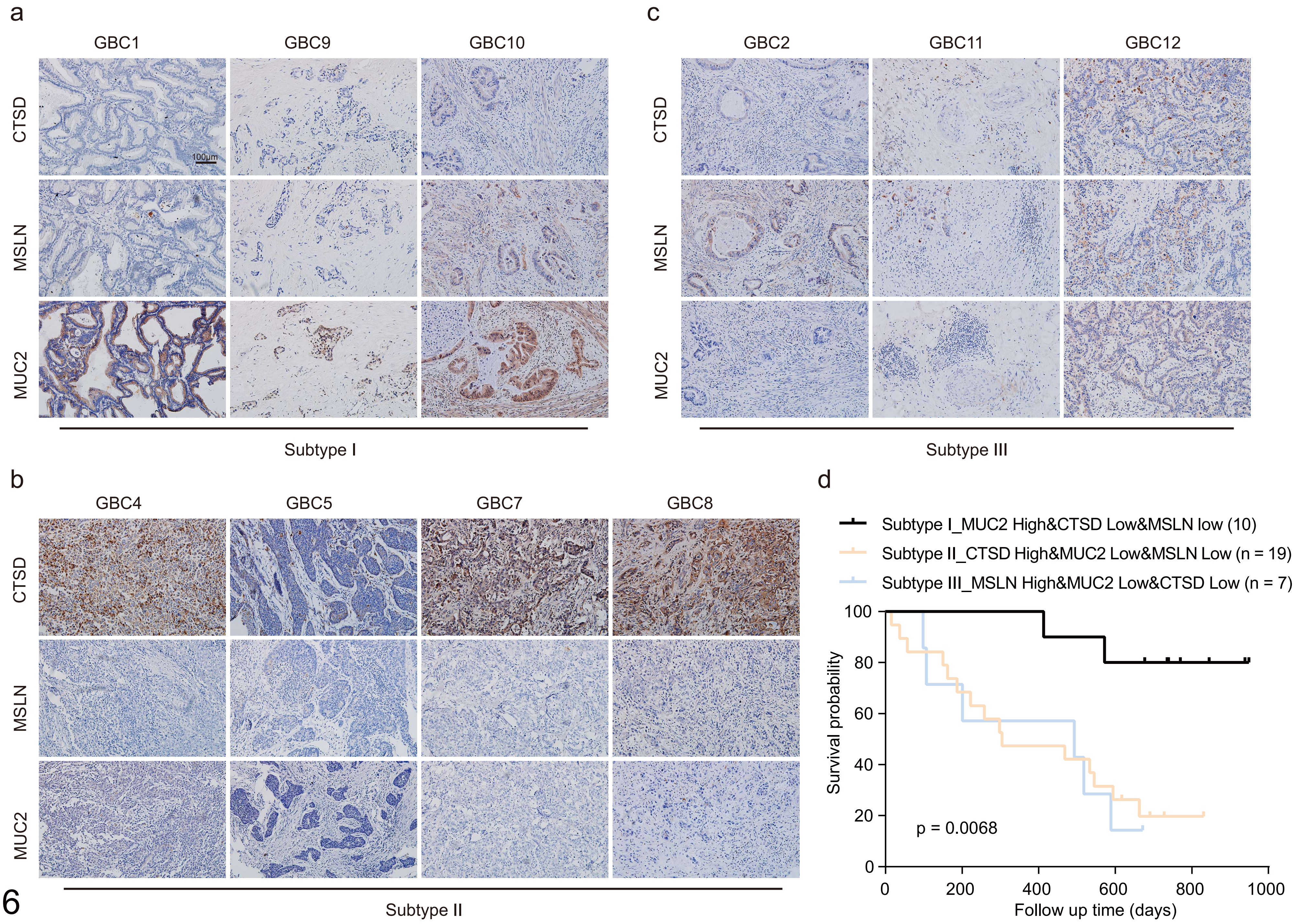

a
GBC1
GBC9
GBC10
CTSD
MSLN
MUC2
100μm
Subtype I
c
GBC2
GBC11
GBC12
CTSD
MSLN
MUC2
Subtype III
b
GBC4
GBC5
GBC7
GBC8
CTSD
MSLN
MUC2
Subtype II
d
Subtype I_MUC2 High&CTSD Low&MSLN low (10)
Subtype II_CTSD High&MUC2 Low&MSLN Low (n = 19)
Subtype III_MSLN High&MUC2 Low&CTSD Low (n = 7)
Survival probability
100
80
60
40
20
0
p = 0.0068
0
200
400
600
800
1000
Follow up time (days)

Fig.S6

Supplementary Fig. S6. IHC staining images of subtype-specific markers (subtype I: MUC2; subtype II: CTSD; subtype III: MSLN) across GBC samples in our scRNA-seq cohort. a Images showing $MUC2^{+}CTSD^{-}MSLN^{-}$ staining among samples of GBC1, GBC9, and GBC10, respectively. **b** Images showing $CTSD^{+}MUC2^{-}MSLN^{-}$ staining among samples of GBC4, GBC5, GBC7, and GBC8, respectively. **c** Images showing $MSLN^{+}MUC2^{-}CTSD^{-}$ staining among samples of GBC2, GBC11, and GBC12, respectively. **d.** Kaplan-Meier curves comparing overall survival between three subtypes of GBC patients. Log-rank test, $P = 0.007$.

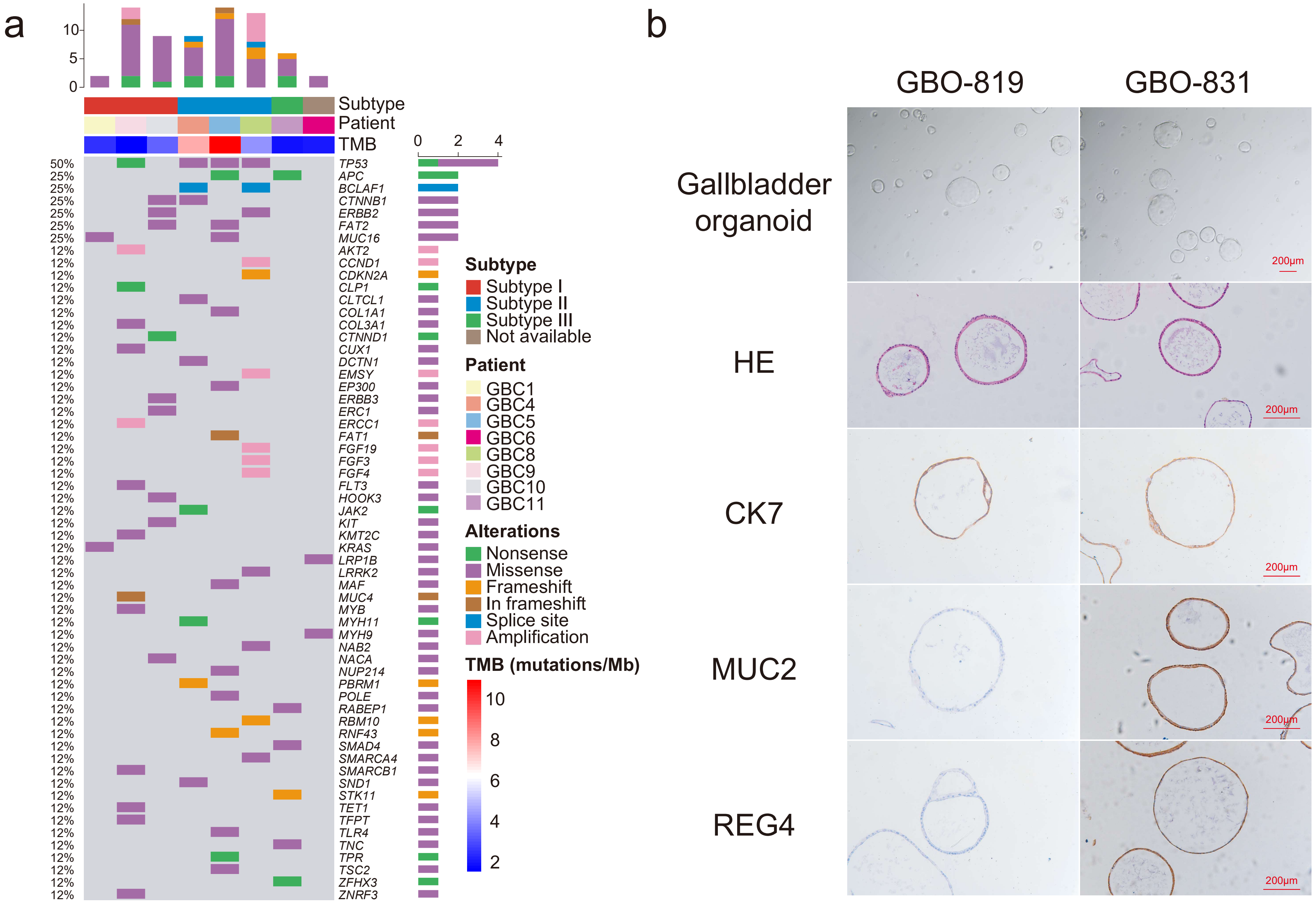
a
10
5
0
Subtype
Patient
TMB
0 2 4
50% TP53
25% APC
25% BCLAF1
25% CTNNB1
25% ERBB2
25% FAT2
25% MUC16
12% AKT2
12% CCND1
12% CDKN2A
12% CLP1
12% CLTCL1
12% COL1A1
12% COL3A1
12% CTNND1
12% CUX1
12% DCTN1
12% EMSY
12% EP300
12% ERBB3
12% ERC1
12% ERCC1
12% FAT1
12% FGF19
12% FGF3
12% FGF4
12% FLT3
12% HOOK3
12% JAK2
12% KIT
12% KMT2C
12% KRAS
12% LRP1B
12% LRRK2
12% MAF
12% MUC4
12% MYB
12% MYH11
12% MYH9
12% NAB2
12% NACA
12% NUP214
12% PBRM1
12% POLE
12% RABEP1
12% RBM10
12% RNF43
12% SMAD4
12% SMARCA4
12% SMARCB1
12% SND1
12% STK11
12% TET1
12% TFPT
12% TLR4
12% TNC
12% TPR
12% TSC2
12% ZFHX3
12% ZNRF3
Subtype
Subtype I
Subtype II
Subtype III
Not available
Patient
GBC1
GBC4
GBC5
GBC6
GBC8
GBC9
GBC10
GBC11
Alterations
Nonsense
Missense
Frameshift
In frameshift
Splice site
Amplification
TMB (mutations/Mb)
10
8
6
4
2
b
GBO-819
GBO-831
Gallbladder organoid
HE
CK7
MUC2
REG4
200μm

Fig.S7

Supplementary Fig. S7. Genomic alterations of GBC samples in our scRNA-seq cohort and the establishment of gallbladder organoids from patients with chronic cholecystitis. a Whole-exome sequencing (WES) revealing distributions of most frequently altered (> 10% of the cohort) oncogenes and tumor suppressor genes (mutations or copy number variations), together with tumor mutation burden (TMB) levels, among eight GBC patients of different mEPC subtypes. Top histograms, frequency of genomic alterations per patient; left column, frequencies of somatic alterations; right column, gene names. **b** Establishment of $MUC2^{-}REG4^{-}$ GBO-819 and $MUC2^{+}REG4^{+}$ GBO-831 gallbladder organoids. First row: bright-field images; second to last rows: H&E and immunohistochemical staining images showing cystic morphology and positive CK7 staining for both organoids. GBO-831 distinctly displayed positive staining of MUC2 and REG4. Scale bars, 200 μm.

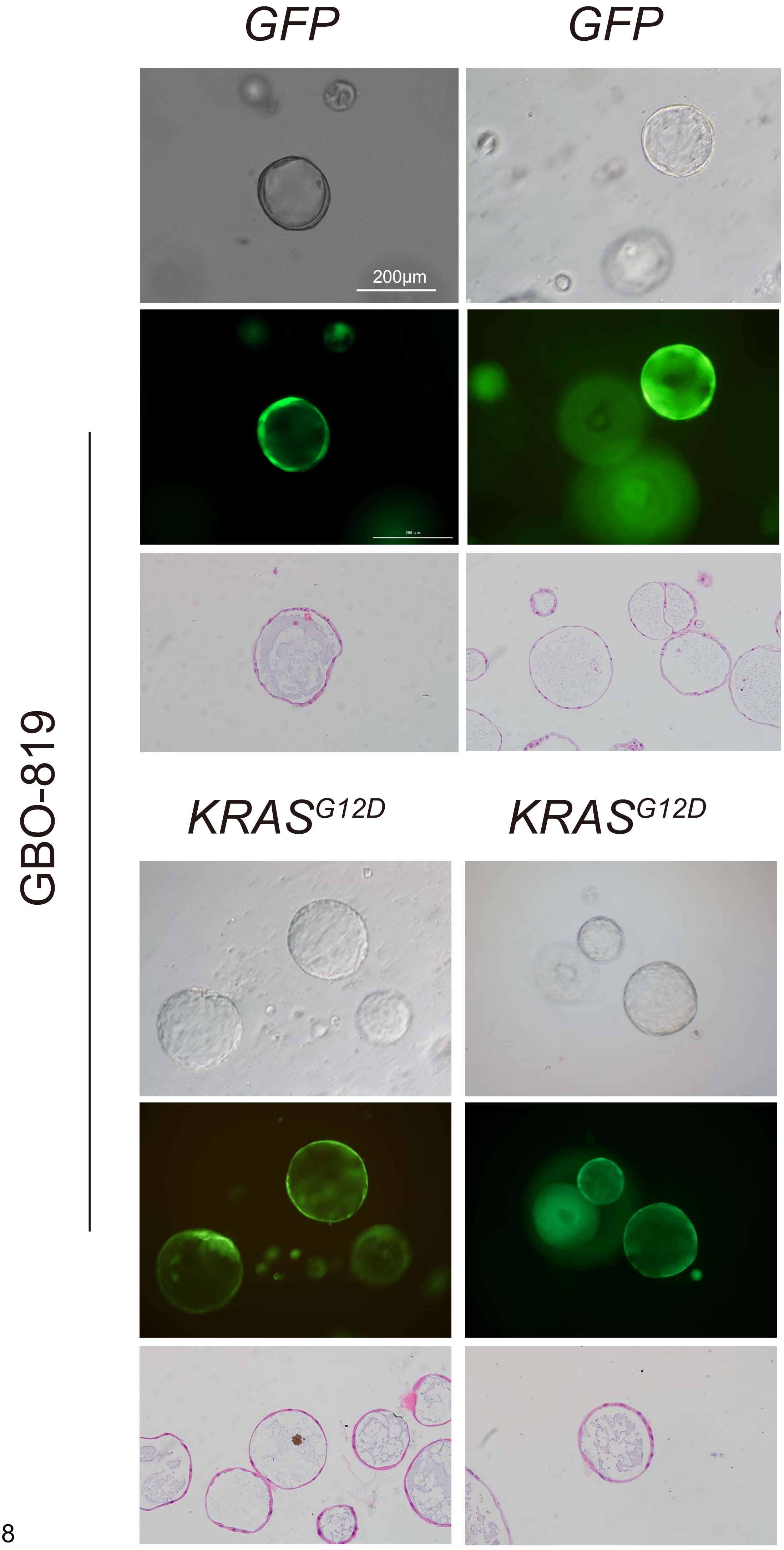

Fig.S8

Supplementary Fig. S8. MUC2^{-}REG4^{-} patient-derived gallbladder organoid (GBO-819) transfected with lentivirus encoding *GFP* (control), or with lentivirus encoding *KRAS*^{G12D}. **a** First to third rows: bright-field, fluorescence, and H&E staining images showing microscopic appearance after two weeks of lenti-*GFP* transfection. Scale bars, 200 μm. **b** First to third rows: bright-field, fluorescence, and H&E staining images showing microscopic appearance after two weeks of lenti-*KRAS*^{G12D} transfection. Scale bars, 200 μm.

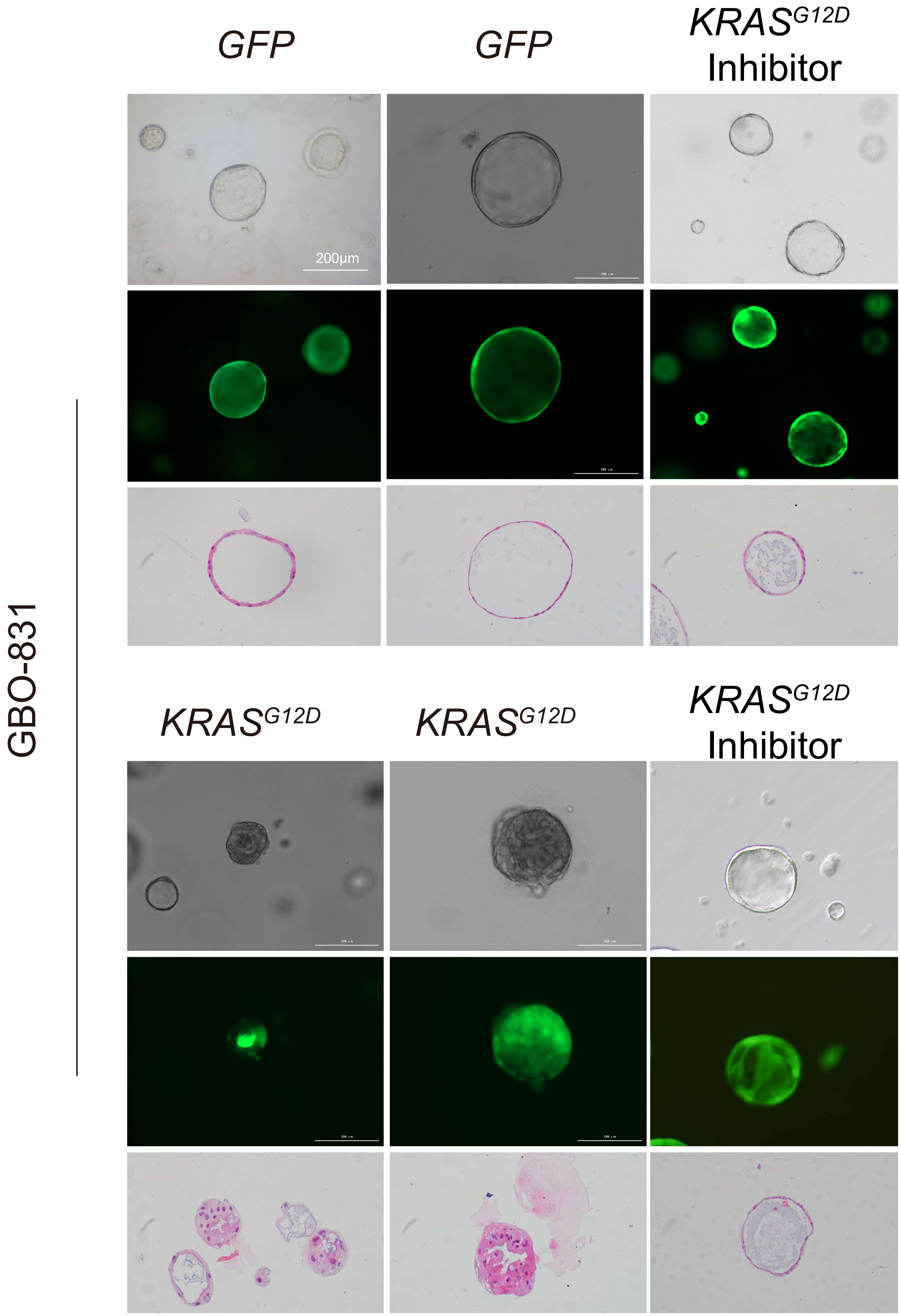

GFP
GFP
KRAS G12D Inhibitor
200μm
KRAS G12D
KRAS G12D
KRAS G12D Inhibitor
GBO-831

Fig.S9

Supplementary Fig. S9. MUC2^{+}REG4^{+} patient-derived gallbladder organoid (GBO-831) transfected with lentivirus encoding *GFP*, or with lentivirus encoding *KRAS*$^{\mathrm{G12D}}$. a First to third rows: bright-field, fluorescence, and H&E staining images showing microscopic appearance after 2 weeks of lenti-*GFP* transfection, with or without treatment of *KRAS*$^{\mathrm{G12D}}$ inhibitor 14 (MedChemExpress LLC, 2 μM). Scale bars, 200 μm. **b** First to third rows: bright-field, fluorescence, and H&E staining images showing microscopic appearance after 2 weeks of lenti-*KRAS*$^{\mathrm{G12D}}$ transfection, with or without treatment of *KRAS*$^{\mathrm{G12D}}$ inhibitor 14 (MedChemExpress LLC, 2 μM). Scale bars, 200 μm.

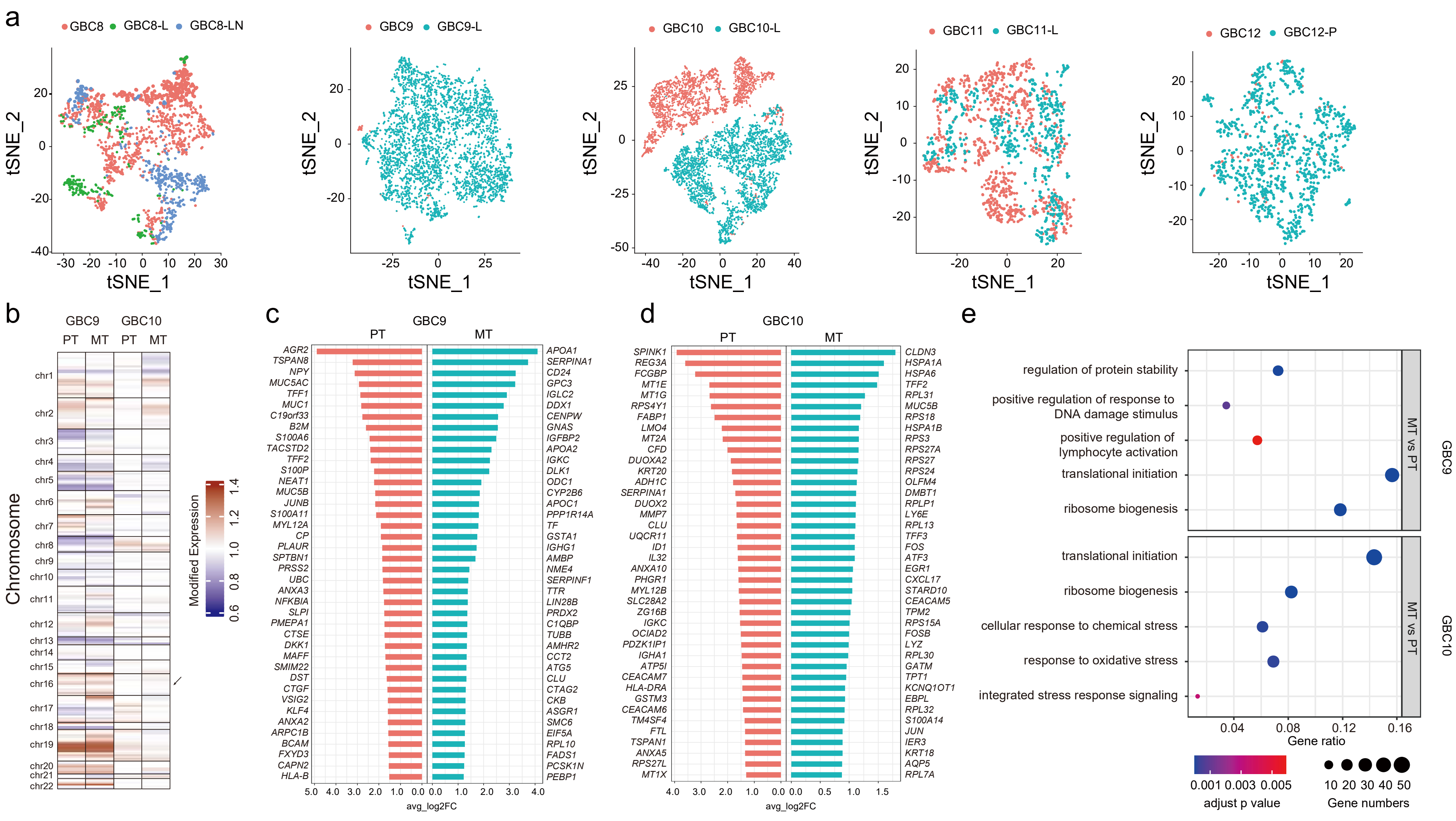
a
GBC8 GBC8-L GBC8-LN
GBC9 GBC9-L
GBC10 GBC10-L
GBC11 GBC11-L
GBC12 GBC12-P
tSNE_2
tSNE_1
b
GBC9 GBC10
PT MT PT MT
Chromosome
chr1
chr2
chr3
chr4
chr5
chr6
chr7
chr8
chr9
chr10
chr11
chr12
chr13
chr14
chr15
chr16
chr17
chr18
chr19
chr20
chr21
chr22
Modified Expression
1.4
1.2
1.0
0.8
0.6
c
GBC9
PT MT
AGR2
TSPAN8
NPY
MUC5AC
TFF1
MUC1
C19orf33
B2M
S100A6
TACSTD2
TFF2
S100P
NEAT1
MUC5B
JUNB
S100A11
MYL12A
CP
PLAUR
SPTBN1
PRSS2
UBC
ANXA3
NFKBIA
SLPI
PMEPA1
CTSE
DKK1
MAFF
SMIM22
DST
CTGF
VSIG2
KLF4
ANXA2
ARPC1B
BCAM
FXYD3
CAPN2
HLA-B
APOA1
SERPINA1
CD24
GPC3
IGLC2
DDX1
CENPW
GNAS
IGFBP2
APOA2
IGKC
DLK1
ODC1
CYP2B6
APOC1
PPP1R14A
TF
GSTA1
IGHG1
AMBP
NME4
SERPINF1
TTR
LIN28B
PRDX2
C1QBP
TUBB
AMHR2
CCT2
ATG5
CLU
CTAG2
CKB
ASGR1
SMC6
EIF5A
RPL10
FADS1
PCSK1N
PEBP1
avg_log2FC
d
GBC10
PT MT
SPINK1
REG3A
FCGBP
MT1E
MT1G
RPS4Y1
FABP1
LMO4
MT2A
CFD
DUOXA2
KRT20
ADH1C
SERPINA1
DUOX2
MMP7
CLU
UQCR11
ID1
IL32
ANXA10
PHGR1
MYL12B
SLC28A2
ZG16B
IGKC
OCIAD2
PDZK1IP1
IGHA1
ATP5I
CEACAM7
HLA-DRA
GSTM3
CEACAM6
TM4SF4
FTL
TSPAN1
ANXA5
RPS27L
MT1X
CLDN3
HSPA1A
HSPA6
TFF2
RPL31
MUC5B
RPS18
HSPA1B
RPS3
RPS27A
RPS27
RPS24
OLFM4
DMBT1
RPLP1
LY6E
RPL13
TFF3
FOS
ATF3
EGR1
CXCL17
STARD10
CEACAM5
TPM2
RPS15A
FOSB
LYZ
RPL30
GATM
TPT1
KCNQ1OT1
EBPL
RPL32
S100A14
JUN
IER3
KRT18
AQP5
RPL7A
avg_log2FC
e
regulation of protein stability
positive regulation of response to DNA damage stimulus
positive regulation of lymphocyte activation
translational initiation
ribosome biogenesis
translational initiation
ribosome biogenesis
cellular response to chemical stress
response to oxidative stress
integrated stress response signaling
MT vs PT
GBC9
MT vs PT
GBC10
Gene ratio
0.04 0.08 0.12 0.16
0.001 0.003 0.005
adjust p value
10 20 30 40 50
Gene numbers

Fig.S10

Supplementary Fig. S10. Comparison of transcriptional profiles between paired primary and metastatic GBC samples. a t-SNE plots showing clustering of primary and metastatic cancer cells across paired samples from GBC patients (n = 5). **B** Heatmap comparing inferred CNV landscapes between PTs and MTs from two patients (GBC9-10). **c-d** Bar plots comparing transcription levels of top-ranking DEGs between PTs and MTs from GBC9 **(c)** and GBC10 **(d)**. **e** Dot plots showing significantly enriched GO pathways for PTs versus paired MTs from two patients (GBC9-10).

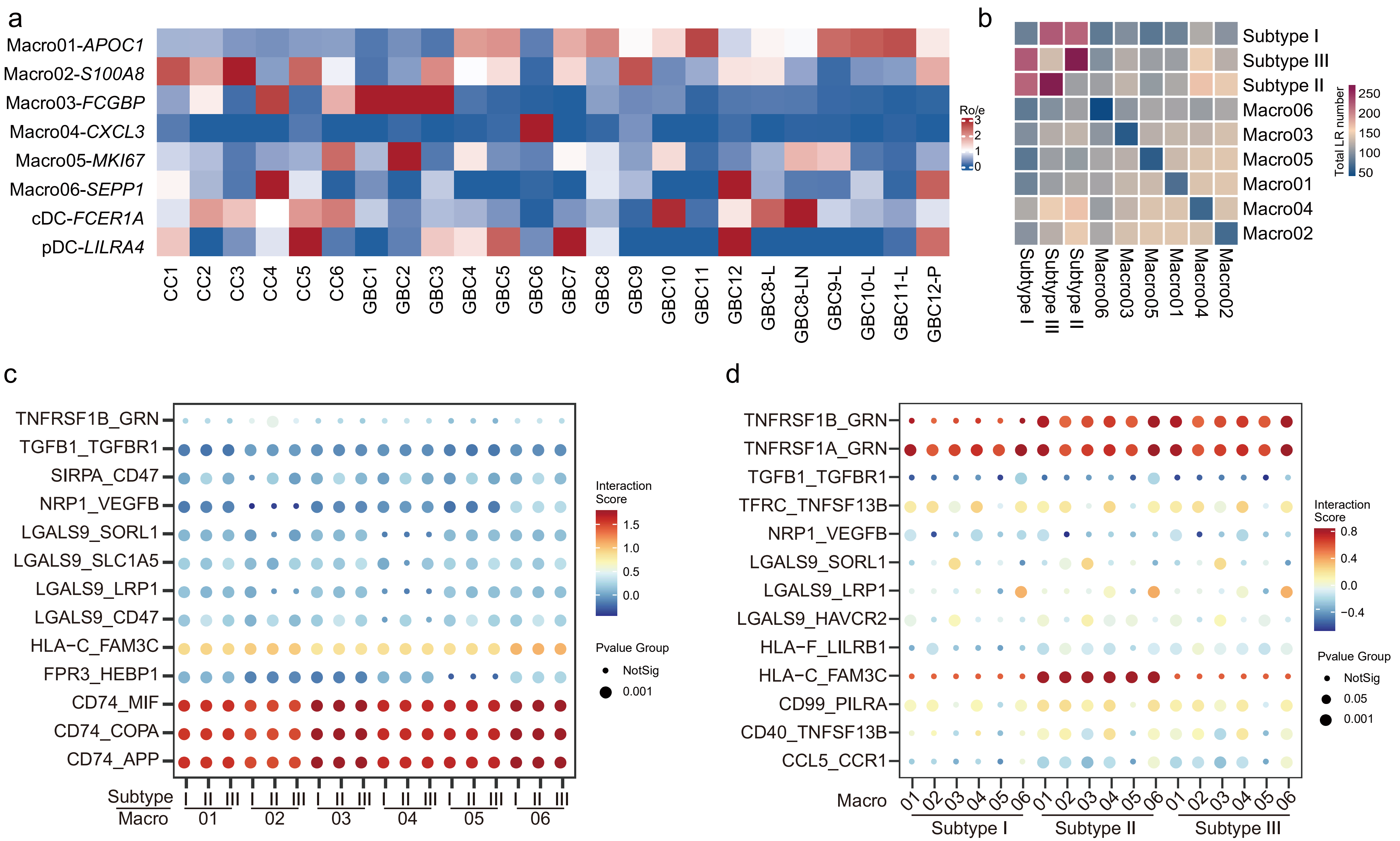
a
Macro01-APOC1
Macro02-S100A8
Macro03-FCGBP
Macro04-CXCL3
Macro05-MKI67
Macro06-SEPP1
cDC-FCER1A
pDC-LILRA4
CC1 CC2 CC3 CC4 CC5 CC6 GBC1 GBC2 GBC3 GBC4 GBC5 GBC6 GBC7 GBC8 GBC9 GBC10 GBC11 GBC12 GBC8-L GBC8-LN GBC9-L GBC10-L GBC11-L GBC12-P
Ro/e
3
2
1
0
b
Subtype I
Subtype III
Subtype II
Macro06
Macro03
Macro05
Macro01
Macro04
Macro02
Total LR number
250
200
150
100
50
c
TNFRSF1B_GRN
TGFB1_TGFBR1
SIRPA_CD47
NRP1_VEGFB
LGALS9_SORL1
LGALS9_SLC1A5
LGALS9_LRP1
LGALS9_CD47
HLA-C_FAM3C
FPR3_HEBP1
CD74_MIF
CD74_COPA
CD74_APP
Subtype
Macro
01 02 03 04 05 06
Interaction Score
1.5
1.0
0.5
0.0
Pvalue Group
NotSig
0.001
d
TNFRSF1B_GRN
TNFRSF1A_GRN
TGFB1_TGFBR1
TFRC_TNFSF13B
NRP1_VEGFB
LGALS9_SORL1
LGALS9_LRP1
LGALS9_HAVCR2
HLA-F_LILRB1
HLA-C_FAM3C
CD99_PILRA
CD40_TNFSF13B
CCL5_CCR1
Macro
Subtype I
Subtype II
Subtype III
Interaction Score
0.8
0.4
0.0
−0.4
Pvalue Group
NotSig
0.05
0.001

Fig.S11

Supplementary Fig. S11. Distributions of myeloid cell clusters and interactions between macrophage clusters and mEPC subsets. a Heatmap showing the relative abundance of different myeloid cell clusters in each sample. Ro/e > 1 indicates significant enrichment. **b** Heatmap showing the total number of ligand-receptor interactions between macrophage clusters and mEPC subsets (Subtype I-III). **c-d** Bubble plots showing representative ligand-receptor pairs between macrophage clusters and mEPC subsets (Subtype I-III). Dot size indicates *P* value, colored by interaction score.

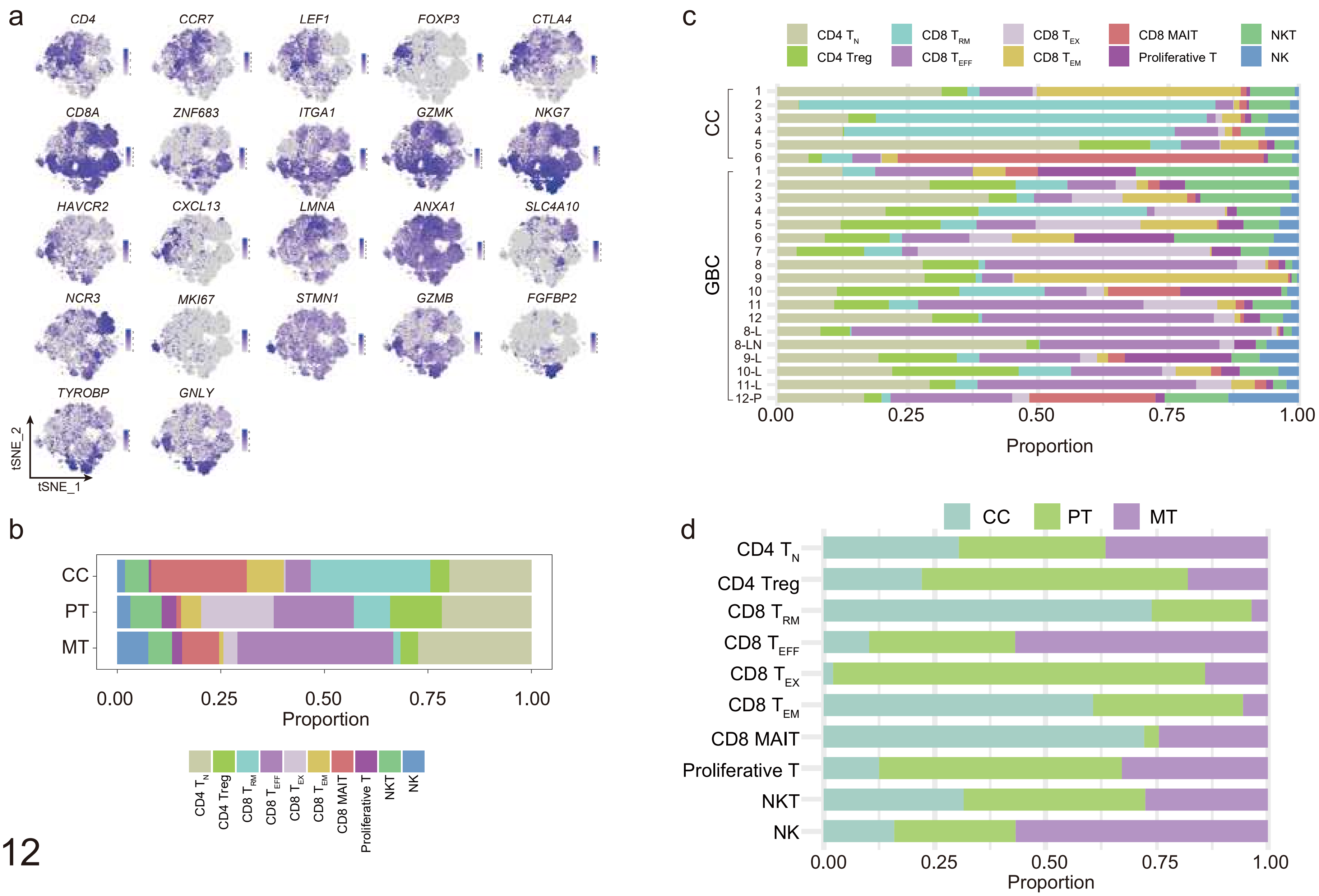
a
CD4
CCR7
LEF1
FOXP3
CTLA4
CD8A
ZNF683
ITGA1
GZMK
NKG7
HAVCR2
CXCL13
LMNA
ANXA1
SLC4A10
NCR3
MKI67
STMN1
GZMB
FGFBP2
TYROBP
GNLY
tSNE_2
tSNE_1
b
CC
PT
MT
0.00
0.25
0.50
0.75
1.00
Proportion
CD4 T_N
CD4 Treg
CD8 T_RM
CD8 T_EFF
CD8 T_EX
CD8 T_EM
CD8 MAIT
Proliferative T
NKT
NK
c
CD4 T_N
CD4 Treg
CD8 T_RM
CD8 T_EFF
CD8 T_EX
CD8 T_EM
CD8 MAIT
Proliferative T
NKT
NK
CC
1
2
3
4
5
6
GBC
1
2
3
4
5
6
7
8
9
10
11
12
8-L
8-LN
9-L
10-L
11-L
12-P
0.00
0.25
0.50
0.75
1.00
Proportion
d
CC
PT
MT
CD4 T_N
CD4 Treg
CD8 T_RM
CD8 T_EFF
CD8 T_EX
CD8 T_EM
CD8 MAIT
Proliferative T
NKT
NK
0.00
0.25
0.50
0.75
1.00
Proportion

Fig.S12

Supplementary Fig. S12. Gene signatures and distributions of T Cells and NK cells. a t-SNE plots visualizing color-coded distributions of marker genes for T cells and NK cells. **b** Horizontal bar plots showing the relative abundance of T cell and NK cell subsets across CCs, PTs, and MTs. **c** Horizontal bar plots showing fractions of T cell and NK cell clusters in each sample. **d** Horizontal bar plots showing tissue sources of each T cell and NK cell cluster.

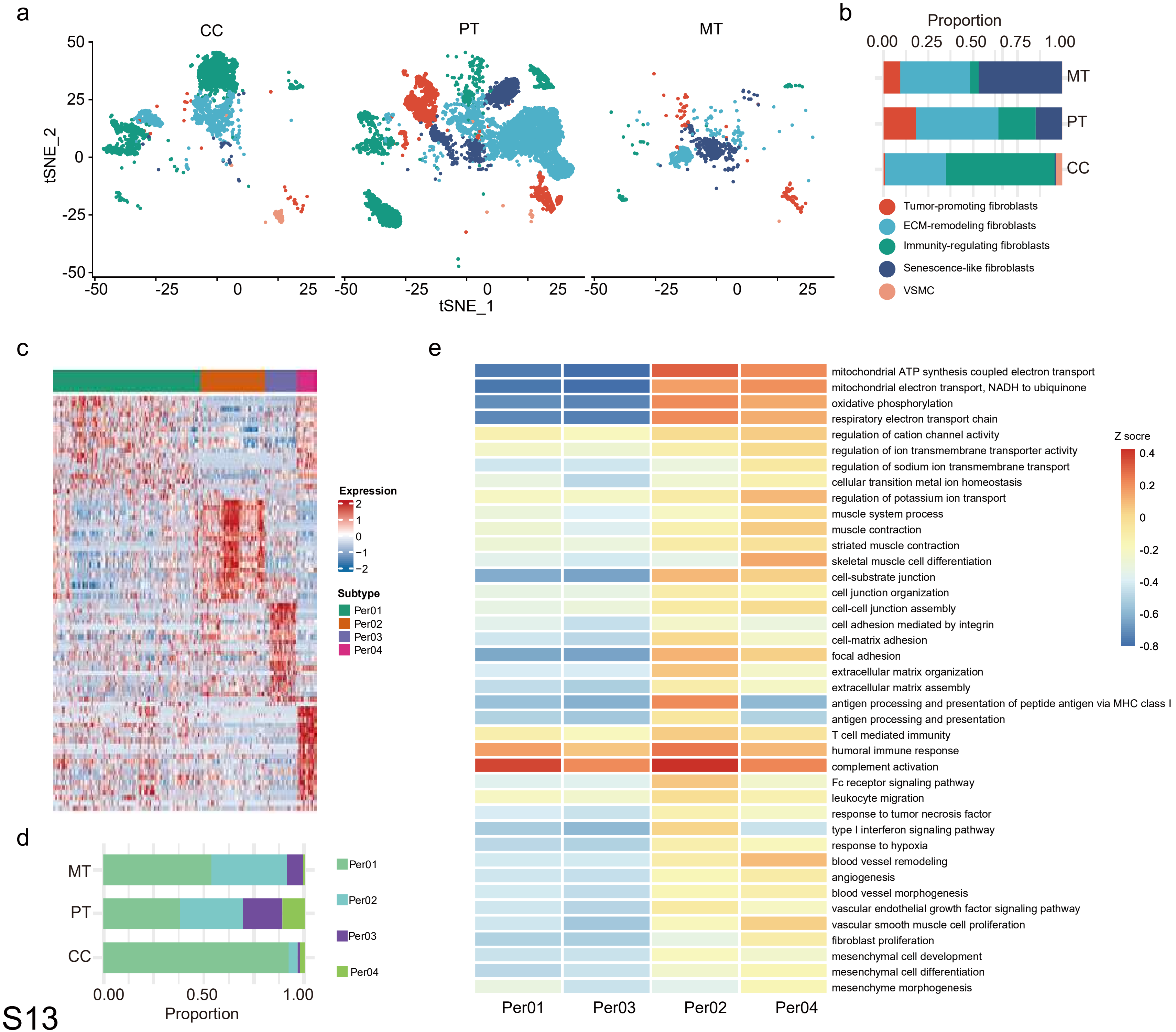
a
CC
PT
MT
tSNE_2
tSNE_1
50
25
0
-25
-50
-50
-25
0
25
b
Proportion
0.00 0.25 0.50 0.75 1.00
MT
PT
CC
Tumor-promoting fibroblasts
ECM-remodeling fibroblasts
Immunity-regulating fibroblasts
Senescence-like fibroblasts
VSMC
c
Expression
2
1
0
−1
−2
Subtype
Per01
Per02
Per03
Per04
d
MT
PT
CC
0.00
0.50
1.00
Proportion
Per01
Per02
Per03
Per04
e
mitochondrial ATP synthesis coupled electron transport
mitochondrial electron transport, NADH to ubiquinone
oxidative phosphorylation
respiratory electron transport chain
regulation of cation channel activity
regulation of ion transmembrane transporter activity
regulation of sodium ion transmembrane transport
cellular transition metal ion homeostasis
regulation of potassium ion transport
muscle system process
muscle contraction
striated muscle contraction
skeletal muscle cell differentiation
cell-substrate junction
cell junction organization
cell-cell junction assembly
cell adhesion mediated by integrin
cell-matrix adhesion
focal adhesion
extracellular matrix organization
extracellular matrix assembly
antigen processing and presentation of peptide antigen via MHC class I
antigen processing and presentation
T cell mediated immunity
humoral immune response
complement activation
Fc receptor signaling pathway
leukocyte migration
response to tumor necrosis factor
type I interferon signaling pathway
response to hypoxia
blood vessel remodeling
angiogenesis
blood vessel morphogenesis
vascular endothelial growth factor signaling pathway
vascular smooth muscle cell proliferation
fibroblast proliferation
mesenchymal cell development
mesenchymal cell differentiation
mesenchyme morphogenesis
Per01
Per03
Per02
Per04
Z socre
0.4
0.2
0
-0.2
-0.4
-0.6
-0.8

Fig.S13

Supplementary Fig. S13. Distribution of fibroblast subtypes and characterization of pericytes. a t-SNE plot visualizing the distribution of fibroblast subsets across CCs, PTs, and MTs. Five subsets were colored in line with **b**. **b** Horizontal bar plots showing fractions of different fibroblast subsets across CCs, PTs, and MTs. **c** Heatmap showing specific expression profiles for each pericyte cluster. **d** The relative abundance of pericytes subsets across CCs, PTs, and MTs. **e** Heatmap showing differentially enriched GO terms across four pericyte clusters, based on GSVA analysis.

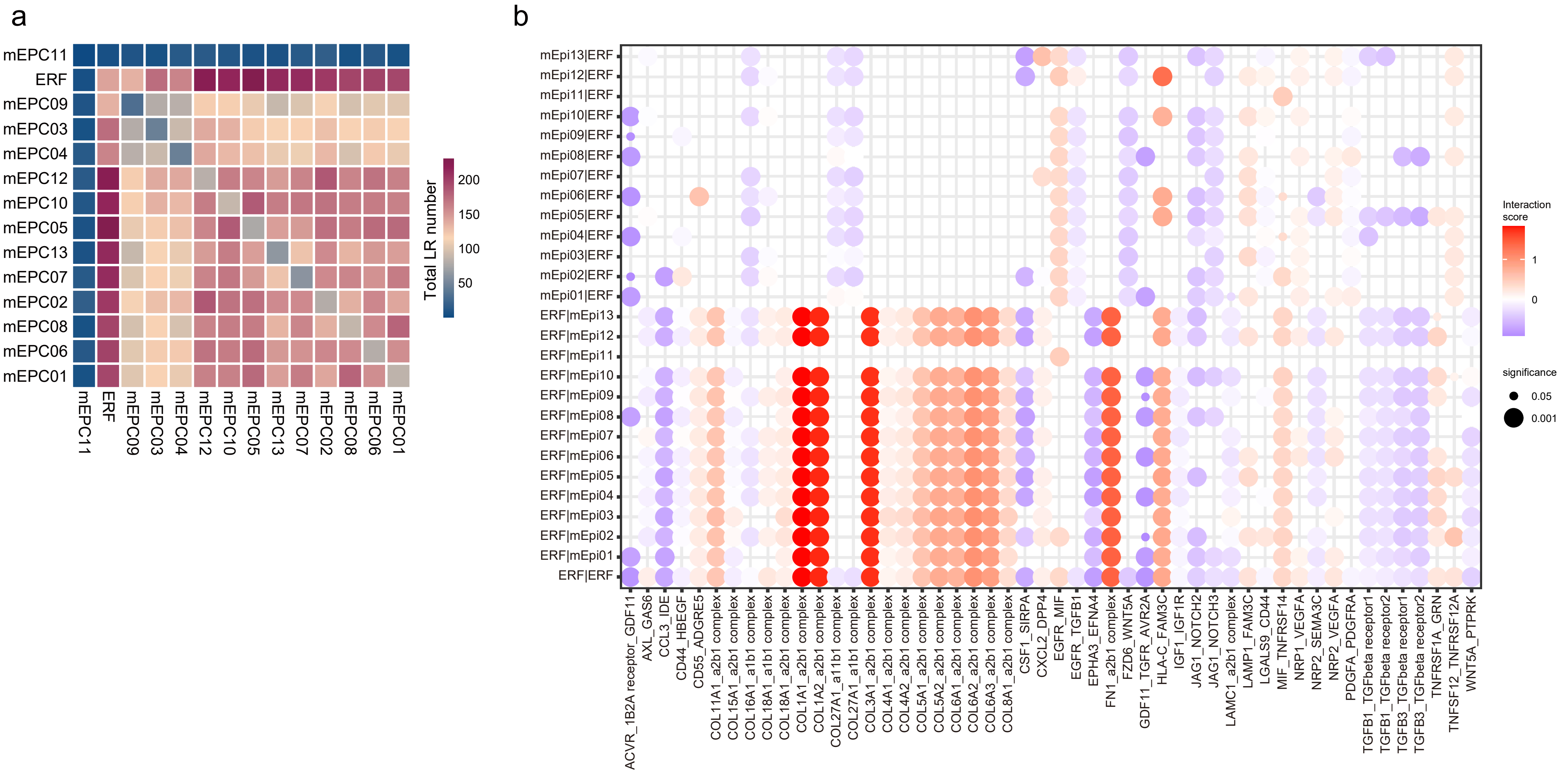
a
mEPC11
ERF
mEPC09
mEPC03
mEPC04
mEPC12
mEPC10
mEPC05
mEPC13
mEPC07
mEPC02
mEPC08
mEPC06
mEPC01
Total LR number
200
150
100
50
b
mEpi13|ERF
mEpi12|ERF
mEpi11|ERF
mEpi10|ERF
mEpi09|ERF
mEpi08|ERF
mEpi07|ERF
mEpi06|ERF
mEpi05|ERF
mEpi04|ERF
mEpi03|ERF
mEpi02|ERF
mEpi01|ERF
ERF|mEpi13
ERF|mEpi12
ERF|mEpi11
ERF|mEpi10
ERF|mEpi09
ERF|mEpi08
ERF|mEpi07
ERF|mEpi06
ERF|mEpi05
ERF|mEpi04
ERF|mEpi03
ERF|mEpi02
ERF|mEpi01
ERF|ERF
ACVR_1B2A receptor_GDF11
AXL_GAS6
CCL3_IDE
CD44_HBEGF
CD55_ADGRE5
COL11A1_a2b1 complex
COL15A1_a2b1 complex
COL16A1_a1b1 complex
COL18A1_a1b1 complex
COL18A1_a2b1 complex
COL1A1_a2b1 complex
COL1A2_a2b1 complex
COL27A1_a11b1 complex
COL27A1_a1b1 complex
COL3A1_a2b1 complex
COL4A1_a2b1 complex
COL4A2_a2b1 complex
COL5A1_a2b1 complex
COL5A2_a2b1 complex
COL6A1_a2b1 complex
COL6A2_a2b1 complex
COL6A3_a2b1 complex
COL8A1_a2b1 complex
CSF1_SIRPA
CXCL2_DPP4
EGFR_MIF
EGFR_TGFB1
EPHA3_EFNA4
FN1_a2b1 complex
FZD6_WNT5A
GDF11_TGFR_AVR2A
HLA-C_FAM3C
IGF1_IGF1R
JAG1_NOTCH2
JAG1_NOTCH3
LAMC1_a2b1 complex
LAMP1_FAM3C
LGALS9_CD44
MIF_TNFRSF14
NRP1_VEGFA
NRP2_SEMA3C
NRP2_VEGFA
PDGFA_PDGFRA
TGFB1_TGFbeta receptor1
TGFB1_TGFbeta receptor2
TGFB3_TGFbeta receptor1
TGFB3_TGFbeta receptor2
TNFRSF1A_GRN
TNFSF12_TNFRSF12A
WNT5A_PTPRK
Interaction score
1
0
significance
0.05
0.001

Fig.S14

Supplementary Fig. S14. Ligand-receptor interactions between malignant epithelial clusters and ECM-remolding fibroblasts (ERF) based on CellphoneDB analysis. a Heatmap showing the total number of ligand-receptor interactions between malignant epithelial clusters and ERF. **b** Bubble plot showing significant ligand-receptor pairs between malignant epithelial clusters and ERF. Dot size represents *P* value and dot color intensity indicates interaction score.

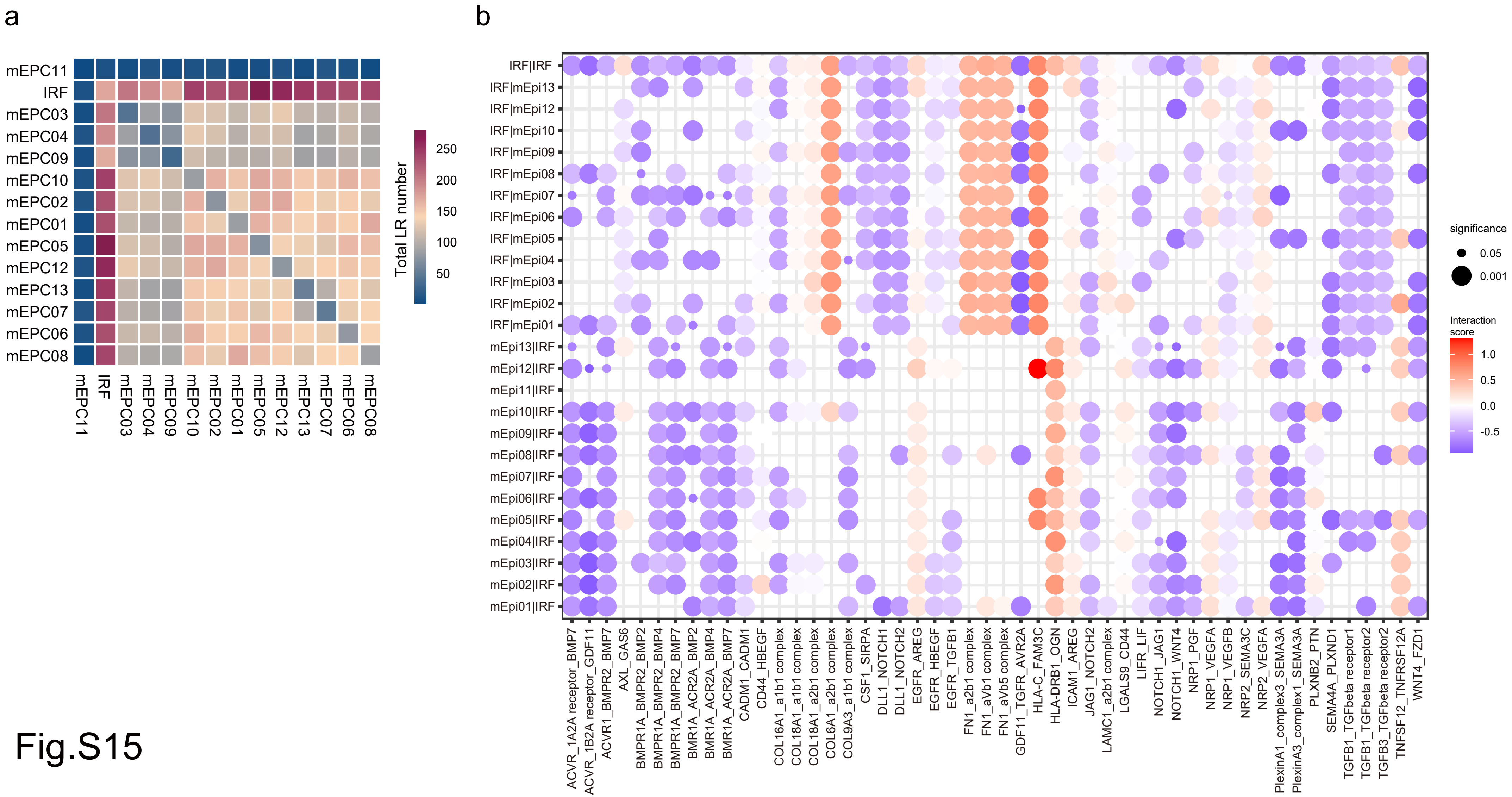
a
b
Total LR number
250
200
150
100
50
mEPC11
IRF
mEPC03
mEPC04
mEPC09
mEPC10
mEPC02
mEPC01
mEPC05
mEPC12
mEPC13
mEPC07
mEPC06
mEPC08
IRF|IRF
IRF|mEpi13
IRF|mEpi12
IRF|mEpi10
IRF|mEpi09
IRF|mEpi08
IRF|mEpi07
IRF|mEpi06
IRF|mEpi05
IRF|mEpi04
IRF|mEpi03
IRF|mEpi02
IRF|mEpi01
mEpi13|IRF
mEpi12|IRF
mEpi11|IRF
mEpi10|IRF
mEpi09|IRF
mEpi08|IRF
mEpi07|IRF
mEpi06|IRF
mEpi05|IRF
mEpi04|IRF
mEpi03|IRF
mEpi02|IRF
mEpi01|IRF
ACVR_1A2A receptor_BMP7
ACVR_1B2A receptor_GDF11
ACVR1_BMPR2_BMP7
AXL_GAS6
BMPR1A_BMPR2_BMP2
BMPR1A_BMPR2_BMP4
BMPR1A_BMPR2_BMP7
BMR1A_ACR2A_BMP2
BMR1A_ACR2A_BMP4
BMR1A_ACR2A_BMP7
CADM1_CADM1
CD44_HBEGF
COL16A1_a1b1 complex
COL18A1_a1b1 complex
COL18A1_a2b1 complex
COL6A1_a2b1 complex
COL9A3_a1b1 complex
CSF1_SIRPA
DLL1_NOTCH1
DLL1_NOTCH2
EGFR_AREG
EGFR_HBEGF
EGFR_TGFB1
FN1_a2b1 complex
FN1_aVb1 complex
FN1_aVb5 complex
GDF11_TGFR_AVR2A
HLA-C_FAM3C
HLA-DRB1_OGN
ICAM1_AREG
JAG1_NOTCH2
LAMC1_a2b1 complex
LGALS9_CD44
LIFR_LIF
NOTCH1_JAG1
NOTCH1_WNT4
NRP1_PGF
NRP1_VEGFA
NRP1_VEGFB
NRP2_SEMA3C
NRP2_VEGFA
PlexinA1_complex3_SEMA3A
PlexinA3_complex1_SEMA3A
PLXNB2_PTN
SEMA4A_PLXND1
TGFB1_TGFbeta receptor1
TGFB1_TGFbeta receptor2
TGFB3_TGFbeta receptor2
TNFSF12_TNFRSF12A
WNT4_FZD1
significance
0.05
0.001
Interaction score
1.0
0.5
0.0
-0.5

Fig.S15

Supplementary Fig. S15. Ligand-receptor interactions between malignant epithelial clusters and immunity-regulating fibroblasts (IRF) based on CellphoneDB analysis. a Heatmap showing the total number of ligand-receptor interactions between malignant epithelial clusters and IRF. **b** Bubble plot showing significant ligand-receptor pairs between malignant epithelial clusters and IRF. Dot size represents *P* value and dot color intensity indicates interaction score.

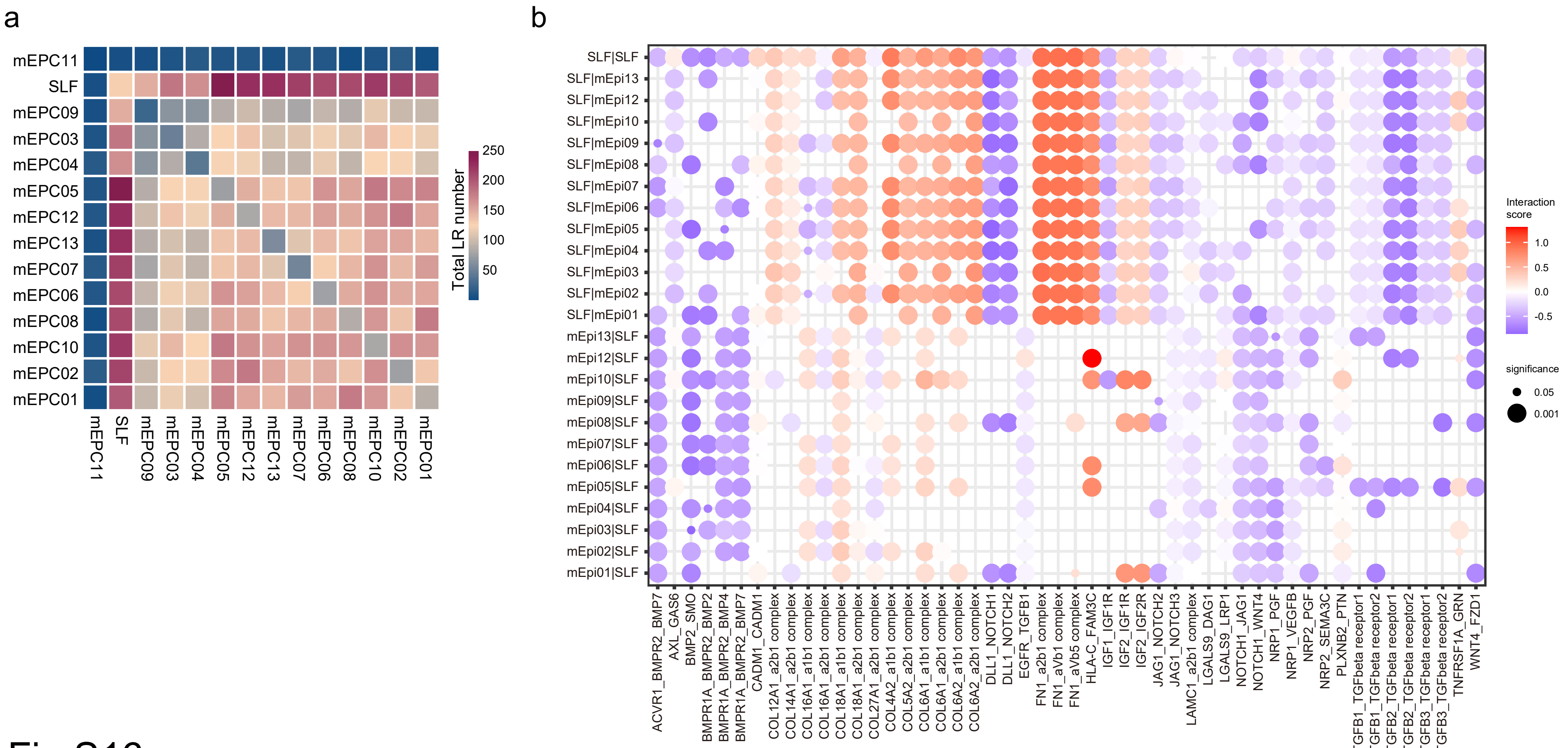
a
b
mEPC11
SLF
mEPC09
mEPC03
mEPC04
mEPC05
mEPC12
mEPC13
mEPC07
mEPC06
mEPC08
mEPC10
mEPC02
mEPC01
Total LR number
250
200
150
100
50
SLF|SLF
SLF|mEpi13
SLF|mEpi12
SLF|mEpi10
SLF|mEpi09
SLF|mEpi08
SLF|mEpi07
SLF|mEpi06
SLF|mEpi05
SLF|mEpi04
SLF|mEpi03
SLF|mEpi02
SLF|mEpi01
mEpi13|SLF
mEpi12|SLF
mEpi10|SLF
mEpi09|SLF
mEpi08|SLF
mEpi07|SLF
mEpi06|SLF
mEpi05|SLF
mEpi04|SLF
mEpi03|SLF
mEpi02|SLF
mEpi01|SLF
ACVR1_BMPR2_BMP7
AXL_GAS6
BMP2_SMO
BMPR1A_BMPR2_BMP2
BMPR1A_BMPR2_BMP4
BMPR1A_BMPR2_BMP7
CADM1_CADM1
COL12A1_a2b1 complex
COL14A1_a2b1 complex
COL16A1_a1b1 complex
COL16A1_a2b1 complex
COL18A1_a1b1 complex
COL18A1_a2b1 complex
COL27A1_a2b1 complex
COL4A2_a1b1 complex
COL5A2_a2b1 complex
COL6A1_a1b1 complex
COL6A1_a2b1 complex
COL6A2_a1b1 complex
COL6A2_a2b1 complex
DLL1_NOTCH1
DLL1_NOTCH2
EGFR_TGFB1
FN1_a2b1 complex
FN1_aVb1 complex
FN1_aVb5 complex
HLA-C_FAM3C
IGF1_IGF1R
IGF2_IGF1R
IGF2_IGF2R
JAG1_NOTCH2
JAG1_NOTCH3
LAMC1_a2b1 complex
LGALS9_DAG1
LGALS9_LRP1
NOTCH1_JAG1
NOTCH1_WNT4
NRP1_PGF
NRP1_VEGFB
NRP2_PGF
NRP2_SEMA3C
PLXNB2_PTN
TGFB1_TGFbeta receptor1
TGFB1_TGFbeta receptor2
TGFB2_TGFbeta receptor1
TGFB2_TGFbeta receptor2
TGFB3_TGFbeta receptor1
TGFB3_TGFbeta receptor2
TNFRSF1A_GRN
WNT4_FZD1
Interaction score
1.0
0.5
0.0
-0.5
significance
0.05
0.001

Fig.S16

Supplementary Fig. S16. Ligand-receptor interactions between malignant epithelial clusters and senescence-like fibroblasts (SLF) based on CellphoneDB analysis. a Heatmap showing the total number of ligand-receptor interactions between malignant epithelial clusters and SLF. **b** Bubble plot showing significant ligand-receptor pairs between malignant epithelial clusters and SLF. Dot size represents *P* value and dot color intensity indicates interaction score.

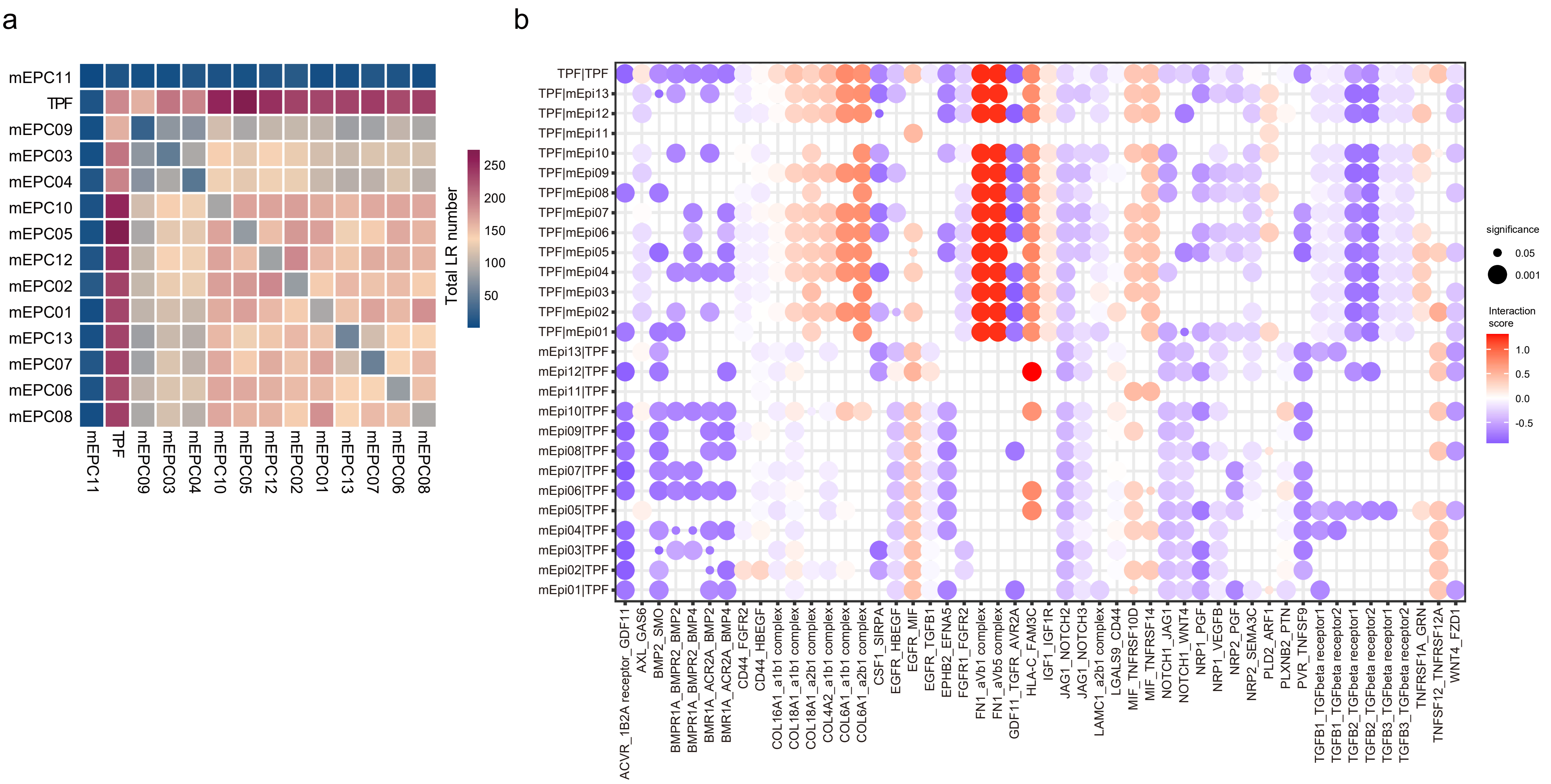
a
mEPC11
TPF
mEPC09
mEPC03
mEPC04
mEPC10
mEPC05
mEPC12
mEPC02
mEPC01
mEPC13
mEPC07
mEPC06
mEPC08
Total LR number
250
200
150
100
50
b
TPF|TPF
TPF|mEpi13
TPF|mEpi12
TPF|mEpi11
TPF|mEpi10
TPF|mEpi09
TPF|mEpi08
TPF|mEpi07
TPF|mEpi06
TPF|mEpi05
TPF|mEpi04
TPF|mEpi03
TPF|mEpi02
TPF|mEpi01
mEpi13|TPF
mEpi12|TPF
mEpi11|TPF
mEpi10|TPF
mEpi09|TPF
mEpi08|TPF
mEpi07|TPF
mEpi06|TPF
mEpi05|TPF
mEpi04|TPF
mEpi03|TPF
mEpi02|TPF
mEpi01|TPF
ACVR_1B2A receptor_GDF11
AXL_GAS6
BMP2_SMO
BMPR1A_BMPR2_BMP2
BMPR1A_BMPR2_BMP4
BMR1A_ACR2A_BMP2
BMR1A_ACR2A_BMP4
CD44_FGFR2
CD44_HBEGF
COL16A1_a1b1 complex
COL18A1_a1b1 complex
COL18A1_a2b1 complex
COL4A2_a1b1 complex
COL6A1_a1b1 complex
COL6A1_a2b1 complex
CSF1_SIRPA
EGFR_HBEGF
EGFR_MIF
EGFR_TGFB1
EPHB2_EFNA5
FGFR1_FGFR2
FN1_aVb1 complex
FN1_aVb5 complex
GDF11_TGFR_AVR2A
HLA-C_FAM3C
IGF1_IGF1R
JAG1_NOTCH2
JAG1_NOTCH3
LAMC1_a2b1 complex
LGALS9_CD44
MIF_TNFRSF10D
MIF_TNFRSF14
NOTCH1_JAG1
NOTCH1_WNT4
NRP1_PGF
NRP1_VEGFB
NRP2_PGF
NRP2_SEMA3C
PLD2_ARF1
PLXNB2_PTN
PVR_TNFSF9
TGFB1_TGFbeta receptor1
TGFB1_TGFbeta receptor2
TGFB2_TGFbeta receptor1
TGFB2_TGFbeta receptor2
TGFB3_TGFbeta receptor1
TGFB3_TGFbeta receptor2
TNFRSF1A_GRN
TNFSF12_TNFRSF12A
WNT4_FZD1
significance
0.05
0.001
Interaction score
1.0
0.5
0.0
-0.5

Fig.S17

Supplementary Fig. S17. Ligand-receptor interactions between malignant epithelial clusters and tumor-promoting fibroblasts (TPF) based on CellphoneDB analysis. a Heatmap showing the total number of ligand-receptor interactions between malignant epithelial clusters and TPF. **b** Bubble plot showing significant ligand-receptor pairs between malignant epithelial clusters and TPF. Dot size represents *P* value and dot color intensity indicates interaction score.
